# Subjective experience of listening to favorite songs and Murottal Al-Qur’an in young adult women: a quantitative EEG case series

**DOI:** 10.3389/fnbeh.2026.1903142

**Published:** 2026-09-24

**Authors:** Kana Khairunnisa, Yusuf Alam Romadhon

**Affiliations:** Faculty of Medicine, Muhammadiyah University of Surakarta, Surakarta, Indonesia

**Keywords:** case series, EEG, favorite song, murottal, quantitative EEG, subjective experience, young adult women

## Abstract

**Introduction:**

Listening to music and Qur’anic murottal is a conscious auditory experience associated with beneficial health outcomes. Both activities share the same sensory modality called audition; however, murottal listening is additionally linked to spiritual belief, whereas music listening is not necessarily so, although both are reported to have favorable effects on health. The subjective, phenomenological aspect of conscious listening has rarely been examined alongside objective neurophysiological measurements.

**Methods:**

In this descriptive case series, four healthy 19-year-old female participants underwent a fixed-order recording session that compared mean alpha-band (8–12 Hz) power during listening (their favorite song and murottal, 3 min each), with mean alpha power during an eyes-closed, resting baseline, expressed as a relative percentage change: (E − B)/B × 100%. This index reflects relative alpha event-related desynchronization/synchronization (ERD/ERS) rather than the classical event-related potential. Heart rate and unstructured subjective reports were collected concurrently.

**Results and conclusion:**

Individual alpha ERD/ERS topography broadly aligned with participants’ self-reported mental states—relaxation, visual imagery, and concentration—during both music and murottal listening, with notable between-subject variability in hemispheric lateralization. Given the small, non-randomized sample and the absence of inferential statistics, these observations are considered as hypothesis-generating rather than generalizable and are intended to inform the design of a larger, counterbalanced study.

## Conscious experience of listening to music and Murottal Al-Qur’an

The conscious experience of listening to music arises from the engagement of distributed neural networks following the perception of auditory and rhythmic features produced by the music (Bayne and Williams, 2023). This series of music-related conscious events further evokes neural processing of meaning creation and aesthetic awareness during listening (Giacosa, 2023). Music can also change a person’s state of consciousness, such as into a shamanic state, through auditory rhythm tuning and certain neurofeedback exercises (Hofstädter, 2023). In addition, music strongly influences an individual’s emotions, thoughts, actions, and cognitive functions. These changes in behavior, for example, affect shopping behavior and strengthen or weaken motivation, so they are used as a part of psychotherapy (Jones et al., 2020). The sound of music is unique and can become a cultural identity, and individual responses have different uniqueness (Wardani et al., 2023; Reybrouck et al., 2021). Listening to music often generates a sense of pleasure, as a reflection of the performance of the reward circuit in the brain (Reybrouck and Eerola, 2022). Music, with its character of rhythms, tones, and pauses of silence when perceived auditorily, triggers various evocations of neural activity in the brain. The resulting evocations include a sense of deep emotion, foreshadowing of horizons, and triggering of relevant memories related to various life events (Belfi, 2023; Mateu et al., 2023).

The verses of the Qur’an recited during murottal murottal are, in principle, auditory experience that engages senses and evokes various neural processing, fostering psychospiritual consciousness of the Qur’anic verses heard (Kannan et al., 2022). This psychospiritual consciousness is created because of previous beliefs, so that this activity of listening to Qur’anic murottal has a transformative power to evoke a sense of catharsis, relaxation, and relief from psychoemotional tension (Weinrich, 2019). The experience of listening to Qur’anic murottal, phenomenologically, is unique, and according to a study, this experience is influenced by gender differences, education levels, and the intensity of a person’s psychospiritual interaction with the Qur’an (Ismail, 2023). Exploration related to the experience of psychospiritual consciousness when listening to Qur’anic murottal shows that the activity of listening to Qur’anic murottal triggers the neural networks involved in empathy, “mentalizing,” and episodic memory. As a result, individuals can perceive visual images in the space of consciousness according to the content of the Qur’anic verses they hear (AlMahrouqi and Mostafa, 2023). Another impact of listening to Qur’anic murottal on coma patients in the intensive care unit can increase their level of consciousness (Naseri-Salahshour et al., 2018). A 2026 study further corroborates the neurobiological basis of this effect, reporting that structured Qur’an recitation is associated with increased alpha-band activity alongside favorable modulations of neurotrophic and stress-related biomarkers (Okur and Bayraktar, 2026).

## Quantitative EEG

Brain neuron cells produce action potentials that summate into rhythmic electrical activity, which can be recorded non-invasively using electroencephalography (Smailovic and Jelic, 2019). These rhythms are categorized into δ – delta (< 4 Hz), θ – theta (4–7 Hz), α – alpha (8–15 Hz), β – beta (16–31 Hz), and γ – gamma (> 31 Hz) bands (Dimitriadis et al., 2019). Quantitative electroencephalography (qEEG) is a non-invasive method of recording brain electrical activity, representing the summation of postsynaptic potentials aligned across activated pyramidal neurons (Kim et al., 2023). Specifically, qEEG applies spectral power analysis across frequency bands as an indicator of regional cortical engagement during specific cognitive tasks (Aubonnet et al., 2023), with temporal resolution on a millisecond scale (Zhao et al., 2023). Distinct mental states are associated with characteristic qEEG signatures; in states of stress and anxiety, for instance, elevated beta-band activity is frequently observed (13–30 Hz), with a corresponding dominance of high beta waves (> 20 Hz) (Leaf et al., 2023). Accordingly, qEEG has previously been used to index brain activity during attention- and error-monitoring tasks (Posada-Quintero et al., 2019) in both healthy and clinical populations (Craik et al., 2019).

A distributed set of brain regions is preferentially active when the brain is not engaged in an externally focused, goal-directed task, referred to as the default mode network (DMN) (Buckner et al., 2008). Comparing spectral band power (e.g., alpha oscillations) between a resting baseline and a task condition quantifies the relative change in oscillatory power. This change is termed event-related desynchronization (ERD) when power decreases, or event-related synchronization (ERS) when power increases, relative to the baseline (Li et al., 2022). This block-averaged relative power change is conceptually distinct from the classical event-related potential (ERP), which denotes time-locked, phase-aligned voltage deflections (e.g., P300, N400) occurring within milliseconds of a discrete, repeated stimulus. Because the present experimental design compares alpha power averaged over a 3-min listening block with a 2-min eyes-closed baseline, the appropriate operational term for our outcome measure is relative alpha ERD/ERS, which is used throughout this manuscript. EEG recordings also show a well-documented right–left asymmetry, more pronounced for verbal than visual processing in the temporoparietal region (Ehrlichman and Wiener, 1980), commonly attributed to hemispheric/hand dominance (Butler and Glass, 1974).

Although explicitly stating gender differences, data presented in a volumetric study of the hippocampus showed that there are differences in the mean volume of both the hippocampus as a whole and its constituent components (Usugi et al., 2020).

## Quantitative EEG and listening to music and Murottals in the female gender

Listening to the auditory recitation of the Qur’an from others, commonly called Al-Qur’an murottal, has a long-standing tradition as a therapeutic modality for promoting psychological well-being among Muslim populations. The acoustic structure of the Qur’ān exhibits inherent rhythmic and aesthetic qualities that evoke psychospiritual tranquility, with favorable effects on mental health (Samhani et al., 2022; Ismail et al., 2023). Active engagement with the Qur’ān through memorization, periodic recitation, and listening has been linked to improvements in sleep quality, blood pressure regulation, and other physical and mental health biomarkers (Che Wan Mohd Rozali et al., 2022), including reduced anxiety during labor (Irmawati et al., 2020) and significant reductions in heart rate and blood pressure following surgery under general anesthesia, as reported in a case study from Universitas Muhammadiyah Surakarta (Nafi’ah et al., 2016). Listening to Qur’anic recitation has been associated with a global decrease in EEG band power across frontal and temporal regions, which are interpreted as neurofunctional correlates of enhanced emotion regulation and relaxation (Ismail et al., 2022; Hamzah et al., 2023). A 2025 systematic review of 22 EEG studies corroborates this pattern, reporting a consistent association between Qur’an listening and increased alpha- and theta-band power linked to meditative and relaxed states, albeit with substantial heterogeneity across methodological designs (Majidi and Rajabi-Tavakkol, 2025).

Listening to music carries an inherent rhythmicity tailored to its intended use (Kannan et al., 2022), and accumulating EEG evidence continues to associate music listening reliably induces relaxed, alpha-dominant oscillatory states, with genre-dependent regional topographical differences (El Sayed et al., 2025). Consistent with this, an investigation at Universitas Muhammadiyah Surakarta demonstrated that a 15 min session of culturally familiar relaxing music (traditional Tingkilan Kutai music) produced a significant reduction in perceived stress among students prior to a high-stakes academic examination (Azzahra et al., 2023). Nonetheless, neuroimaging and electrophysiological findings regarding genre-specific neural substrates of remain partially inconclusive (Majno, 2023). Music listening also produces emotional–cognitive responses that may differ by sex; one study reported higher average EEG frequency-band responses in women than men (Goshvarpour, 2019).

Some differences in the characteristics of the female brain compared to men include a thicker cortex in the majority of areas of the brain. Women tend to have a smaller brain volume and lower motor performance than men (Andrushko et al., 2023). A systematic review found that, in general, men and women share more similarities. However, women consistently excel in auditory processing of phonetic cues during speech perception and production (Sato, 2020). In terms of auditory processing, women are stronger than men when in a passive listener position, as found in a study examining event-related potentials (ERP) (Berchicci et al., 2021). Another difference found in qEEG studies is in terms of working memory. Brain regions involved in working memory among men were more prominent in the posterior dipole (occipital, parietal, and temporal), while in women, they were in the frontal cortex structures (Mayorova et al., 2023). Women’s voices, as sound stimuli that evoke responses in related brain areas, are stronger than men (Shklovsky et al., 2019). In terms of cerebral blood flow in certain regions, there are some differences. Blood flow in the right precuneus, left superior temporal gyrus, left inferior frontal gyrus, left cerebellar tonsil, and right medial temporal gyrus is higher in women than men. Differences in glucose metabolism speed between certain regions of men and women were also found (Shin et al., 2021). The thickness of the left Heschl’s gyrus was greater in women compared to men. This finding implies that biological factors of sex are involved in the neural network of speech production (De Lima Xavier et al., 2019). Listening to music and murottal, to some extent, both evoke calmness (Hamzah et al., 2023). Therefore, the discussion of differences in the prefrontal cortex and amygdala (Alexander and Myles, 2022) serves as a reference point for discussing differences in male and female responses to Qur’anic music or murottal stimuli in this study. Neuroimaging studies show that men are more efficient at regulating emotions than women. This reflects lower prefrontal cortex activity levels and the same amygdala activity in men. However, this sex difference depends on the relevant emotion content between the two, as well as emotion generation. Furthermore, this difference is more associated with the rating of the emotional charge involved, where women are more likely to give higher ratings to negatively emotionally charged events (Min et al., 2023).

## Observation method

Recordings were conducted in a quiet room under natural morning daylight without artificial lighting; participants were seated and asked to open and close their eyes for 2 min each. The initial eyes-closed epoch served as the pre-stimulus baseline, followed by 3 min of listening to a favorite song. A 5 min silent washout period followed, after which a second 2-min eyes-closed baseline was recorded, followed by 3 min of listening to familiar music or murottal Al-Qur’an. This order—favorite song always preceding the familiar music/murottal condition—was identical and fixed for all four participants; it was not randomized or counterbalanced, a methodological limitation addressed in the Discussion. Because a fresh baseline was recorded immediately before each listening condition, the baseline heart rate and alpha power reported for the song condition and for the murottal/familiar-music condition of the same participant are independent measurements taken at different points in the session, which accounts for any difference between the two baseline values reported for a given participant. The electroencephalogram was recorded continuously with a Neurology EEG device™ (Canada), 20 Ag/AgCl electrodes (international 10–20 system), ipsilateral-ear reference, impedance < 5 kΩ, filter settings of 0.53–35 Hz (low cut–high cut) with notch filter on, sensitivity 7 μV/mm, and paper/recording speed 30 mm/s, as documented in the original instrument printouts retained for each participant. Quantitative spectral power analysis (FFT relative power) and the generation of topographic maps were performed using qEEG analysis software from Neurology EEG device (TM), Applied NeuroScience, Inc., Canada. This analysis was applied to the raw recordings exported from the acquisition system. Artifact-free epochs from the baseline and listening conditions were selected for analysis by visual inspection, with alpha activity defined as 8–12 Hz and averaged per electrode. The percentage change in mean alpha power during listening (E) relative to the mean alpha power during the immediately preceding baseline (B) was calculated as:

(E−B)/B×100%

Yielding relative alpha event-related desynchronization/synchronization (ERD/ERS): a positive value indicates relative synchronization (ERS), and a negative value indicates relative desynchronization (ERD) (Li et al., 2022). This block-averaged measure is distinct from the classical event-related potential and should not be conflated with it. The analysis was performed at each electrode and presented in tabulated and topographic form. The left electrodes were Fp1, F7, F3, A1, T3, C3, T5, P3, O1; the right electrodes were Fp2, F4, F8, A2, T4, C4, T6, P4, O2; the midline electrodes were Fz, Cz, and Pz. Artifacts considered during recording included eyelid and facial muscle contraction and cardiac electrical activity, identified by visual inspection of the concurrent ECG channel and excluded from the analyzed epochs; a formal automated artifact-rejection pipeline (e.g., independent component analysis) was not applied, which is noted as a limitation below.

## Operational definition

1. Alpha ERD/ERS: the percentage change in mean alpha (8–12 Hz) power during listening compared with the immediately preceding 2-min eyes-closed baseline. Formula: ERD/ERS (%) = (E − B)/B × 100%, where E is the mean alpha power during listening (music or murottal), and B is the mean alpha power at baseline. Instrument: EEG device. Unit: percent. Scale: continuous.
2. Heart rate: the number of heartbeats in a 10-s sampling window, multiplied by six to obtain beats per minute. Unit: beats per minute. Scale: continuous.

## Serial report

Four female participants took part in this case series. For each participant, we report the subjective, first-person account of the listening experience; the heart rate at baseline versus during listening; and the corresponding alpha ERD/ERS topography, presented as a standardized red–blue heatmap (red = ERS/relative increase, blue = ERD/relative decrease). All participants listened to the same murottal recitation, Surat Al-Alaq, recited by Muzammil Hasbullah (95–100 beats per minute); favorite songs were individually selected by each participant.

## Subject 1 (K, 19 years)

The selected favorite song was Shania Twain’s “*You are still the one*”. The number of *beats of the* song ranges from 95 to 103 *beats per minute* (bpm).

## Subjective report—favorite song

*Relaxed, carried away by the pleasant musical atmosphere with the music entitled “you are still the one,” enjoyed carefree, almost fell asleep too, familiar songs because almost every day listened to when playing musical instruments too, there was no sense of discomfort*.

## Alpha ERD/ERS findings—favorite song

Heart rate frequency at baseline was approximately 115 beats per minute and during music listening it was approximately 95 beats per minute (re-derived by calibrated visual QRS counting on the raw ECG trace; see Discussion, Limitations paragraph, and Supporting File 2). The corresponding alpha ERD/ERS topography is shown in Figure 1.

**Figure 1:**
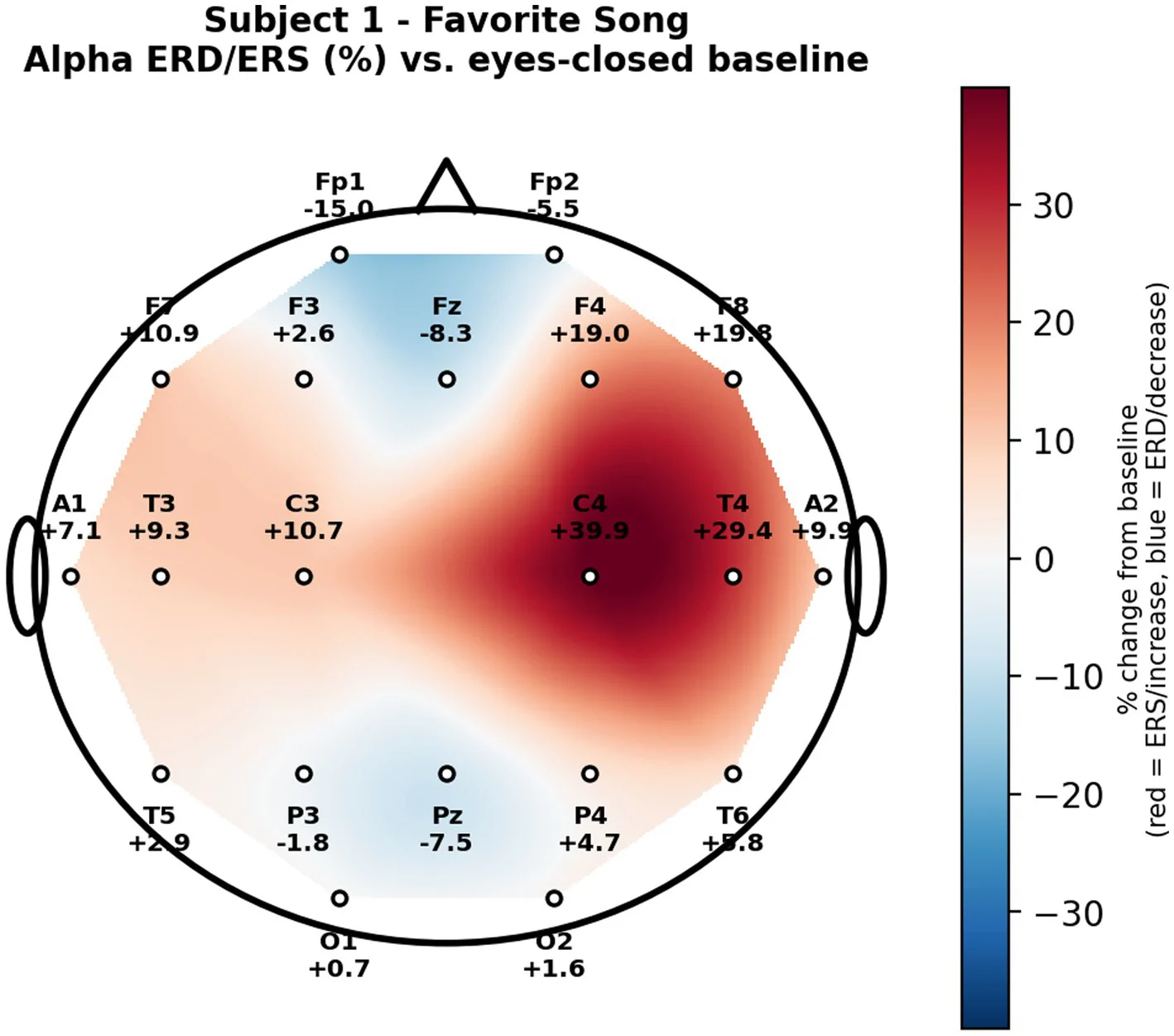
Subject 1 - Favorite Song. Alpha ERD/ERS (%) vs. eyes-closed baseline.

*Alpha ERD/ERS pattern (music listening)*: The cortical response exhibited a predominant, right-lateralized ERS across auricular, temporal, and frontal regions, peaking at electrode C4 (+39.9%), alongside a moderate power increase in occipital and parietal areas. The accompanying reduction in heart rate is consistent with a more relaxed physiological state.

## Subjective report—murottal

*The feeling of being calm, comfortable, and almost falling asleep, but it may be because unconsciously memorizing the passage while remembering the location of the verse on the Qur’an sheet like during murajaah affected the test results*.

Heart rate at baseline was approximately 120 beats per minute, decreasing to approximately 100 beats per minute during Qur’anic murottal listening (quantified *post hoc* by calibrated visual QRS-complex detection on the raw ECG trace; see Discussion, Limitations paragraph, and Supporting File 2). The corresponding alpha ERD/ERS topography is shown in Figure 2.

**Figure 2:**
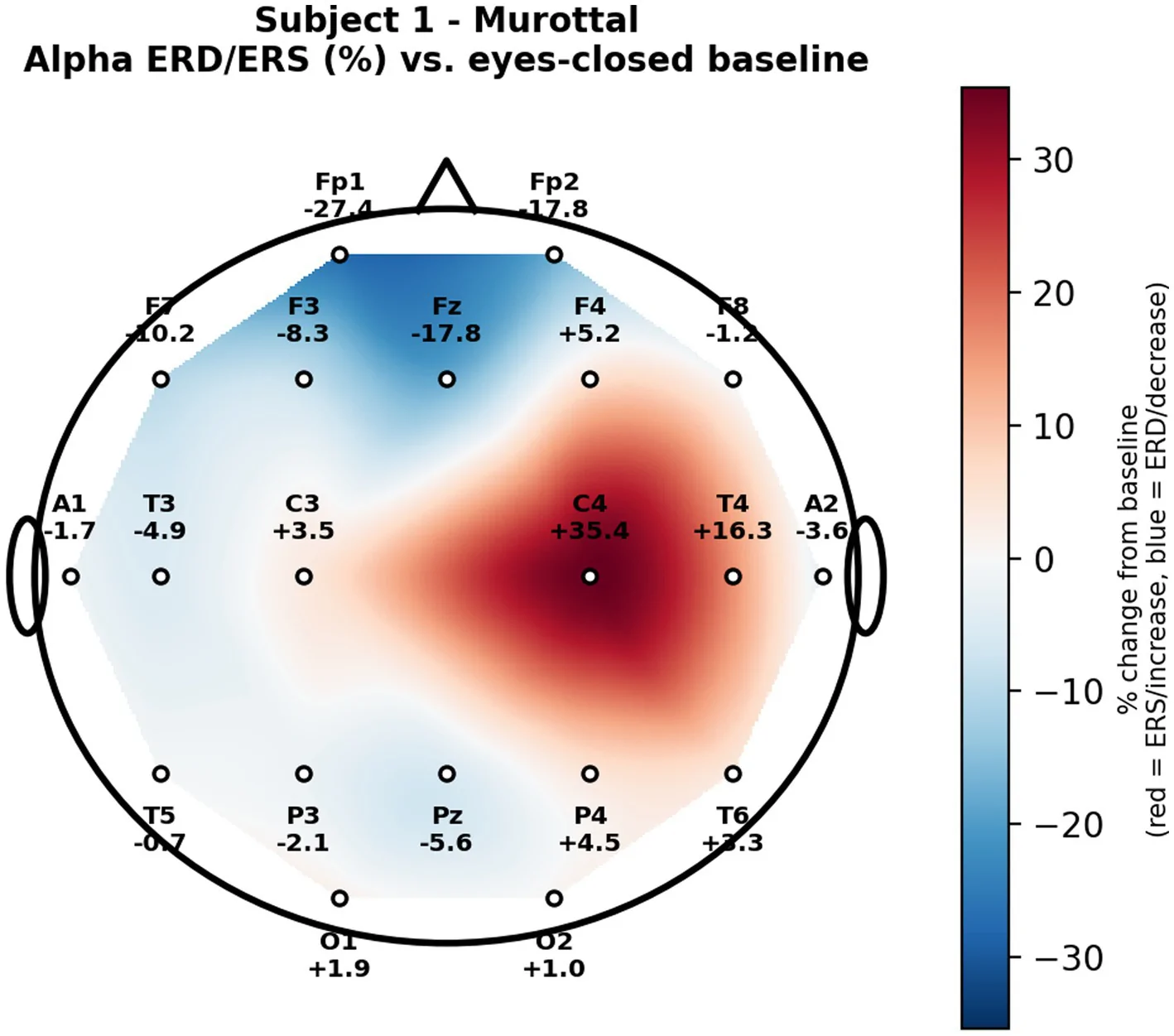
Subject 1 - Murottal. Alpha ERD/ERS (%) vs. eyes-closed baseline.

*Alpha ERD/ERS pattern (Qur’anic murottal listening)*: The cortical response demonstrated a right-lateralized ERS dominant in temporal (C4 + 35.4%) and frontal regions, alongside widespread frontal ERD (Fp1–27.5%, Fz − 17.8%, and Fp2–17.8%). Heart rate decreased relative to baseline, further supporting an autonomic shift toward physiological relaxation.

## Subject 2 (N, 19 years)

The selected favorite song was “Bad” by Wave to Earth with a beat range of 99–103 bpm.

## Subjective report—favorite song

“*When the music started, the slow, soft and light rhythm of the music really spoiled my hearing. From the moment I heard it, my body immediately relaxed and the atmosphere of the room supported the examination. When the first verse was sung, I spontaneously followed the lyrics because it was my favorite song. At the chorus with the lyrics “How could my day be bad when I’m with you, your the only one who makes me smile” my brain suddenly recalled memories of the good old days. As the song progressed my fingers also spontaneously tapped along with the rhythm and tempo of the song.*”

The heart rate at baseline was 60 beats per minute and 66 beats per minute when listening to music. The corresponding alpha ERD/ERS topography is shown in Figure 3.

**Figure 3:**
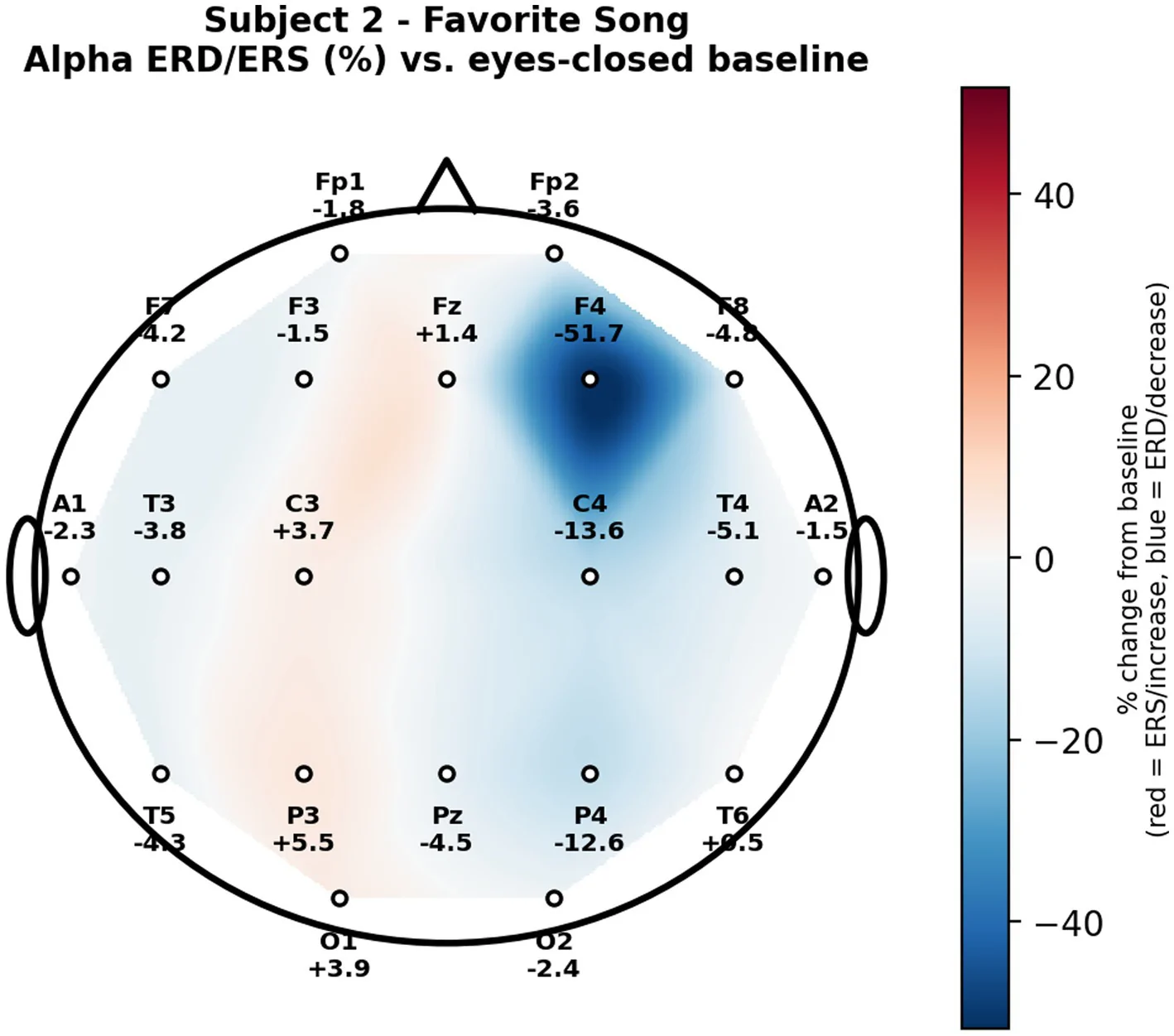
Subject 2 - Favorite Song. Alpha ERD/ERS (%) vs. eyes-closed baseline.

*Alpha ERD/ERS pattern (music listening)*: In contrast to Participant 1, no clear right-hemispheric lateralization was observed; the dominant feature was a strong frontal ERD (F4–51.7%), with mild ERS in occipital (O1 + 3.9%) and parietal (P3 + 5.5%) regions, plausibly reflecting cortical recruitment during autobiographical memory retrieval and visual imagery. The observed increase in heart rate was modest relative to baseline.

## Subjective report—murottal

*I felt the heart-warming chanting of the holy verses when the first verse was recited. The tone was very soft but made me feel safe and peaceful. The verse of the Qur’an that was very familiar to my ears made me enter into the chanting of the verse. It was so soft and peaceful that my body felt like it was floating, enjoying the chanting of the holy verse.*

Heart rate frequency at baseline was 60 beats per minute and at murottal listening was 60 beats per minute. The corresponding alpha ERD/ERS topography is shown in Figure 4.

**Figure 4:**
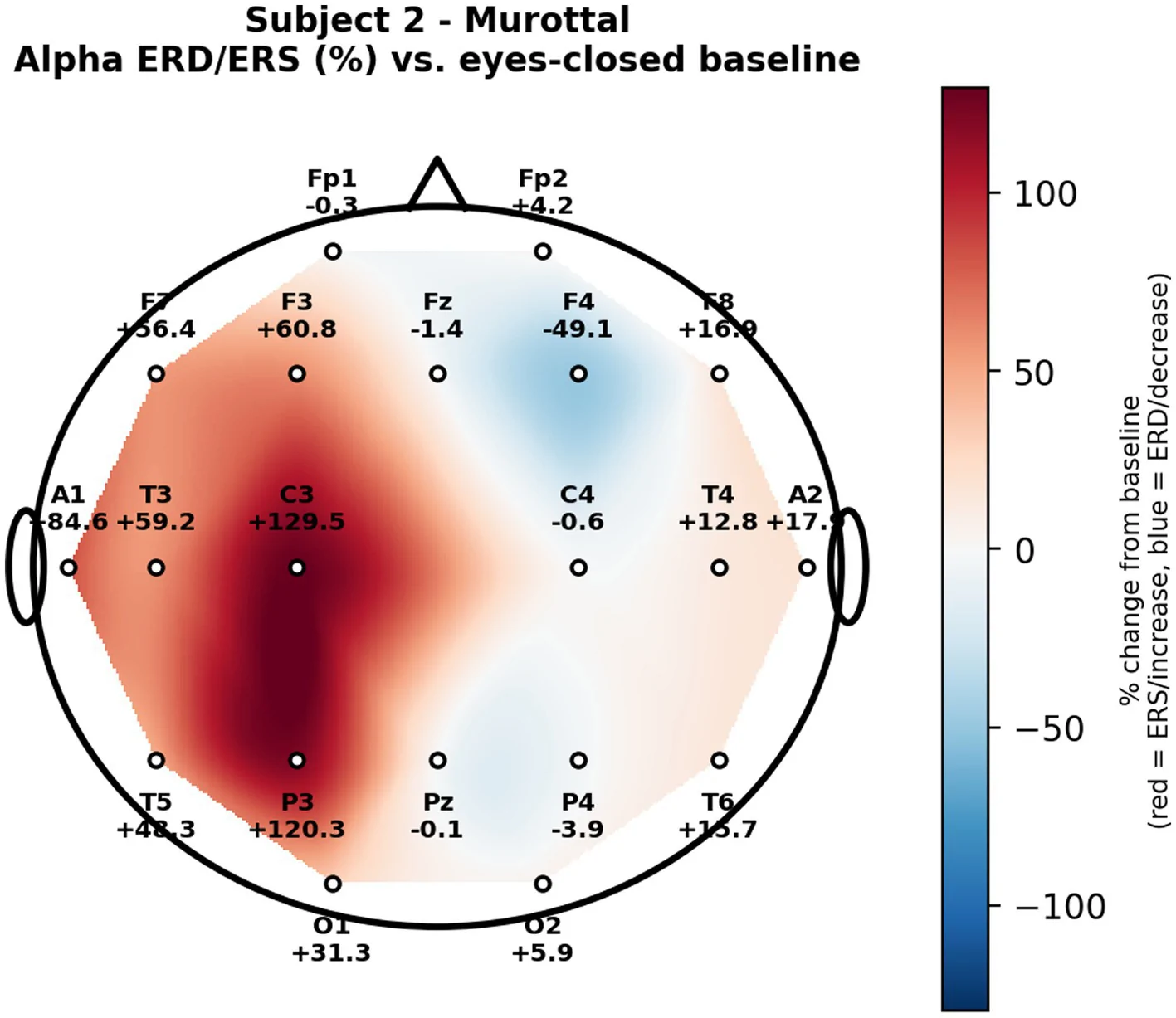
Subject 2 - Murottal. Alpha ERD/ERS (%) vs. eyes-closed baseline.

*Alpha ERD/ERS pattern (murottal)*: The cortical response demonstrated a pronounced left-lateralized ERS dominant in central (C3 + 129.5%), parietal (P3 + 120.3%), and auricular (A1 + 84.6%) regions. Heart rate during Qur’anic murottal listening (60 bpm) was lower than during music listening (66 bpm), consistent with a relatively more relaxed autonomic state.

## Subject 3 (R, 19 years)

The song listened by the participant was “Live While We’re Young” by One Direction, with a beat range of 115–120 bpm.

## Subjective report—favorite song

*“What I listened to was the song ‘Live While We’re Young’ by One Direction. I felt pleasure in my heart and in my imagination because the song is also a cool pop genre. In my imagination, while listening to the song I was on the beach with my friends, going crazy together, riding speedboats and boats in the sea, and enjoying the sunset on the boat, followed by playing music and singing together on the beach with a bonfire and grilling small sausages or fish. After that, I took a car together to the villa. It’s a really fun way to take your mind off things.”*

Heart rate frequency at baseline was 84 beats per minute and during music listening was 78 beats per minute. The corresponding alpha ERD/ERS topography is shown in Figure 5.

**Figure 5:**
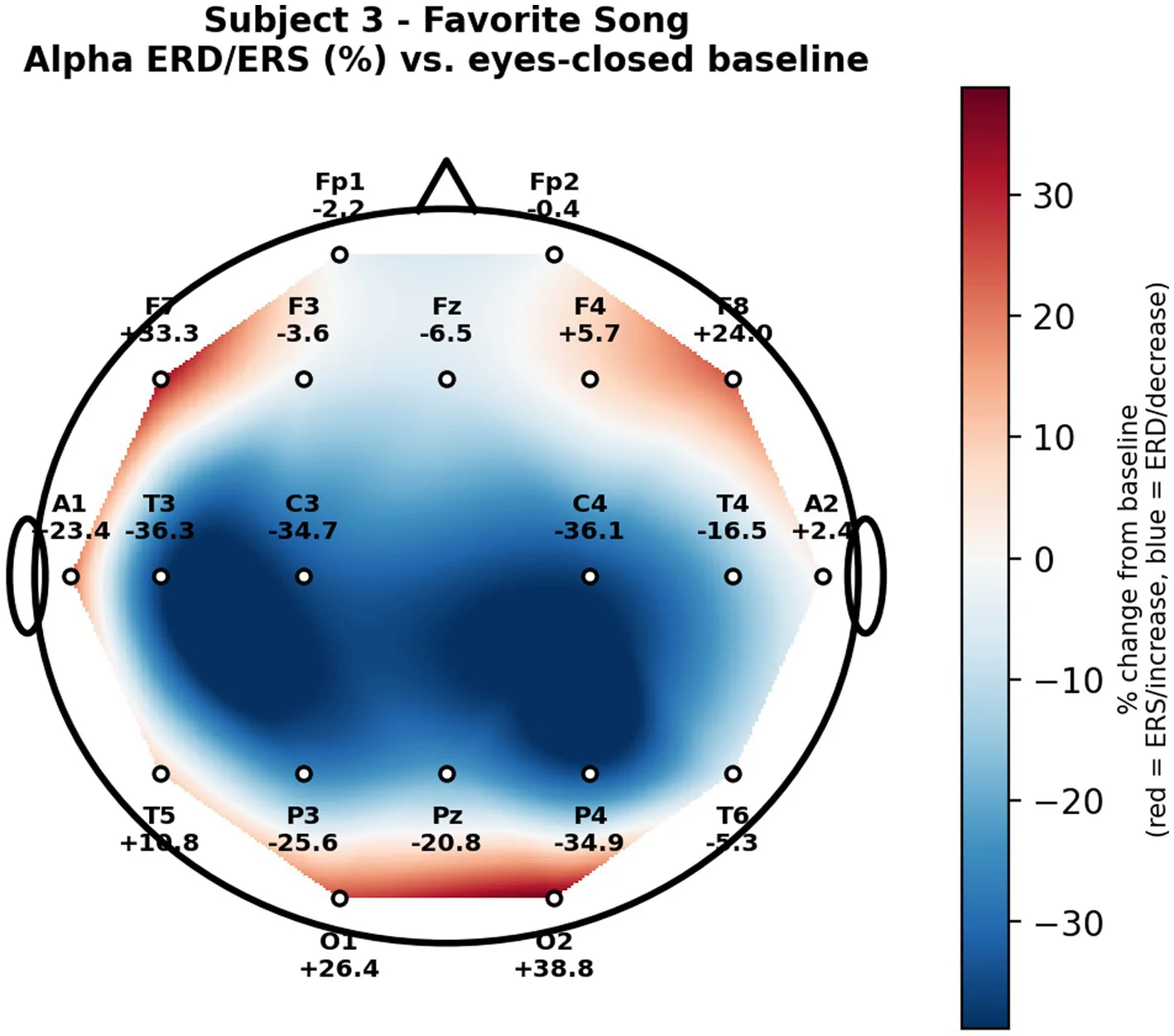
Subject 3 - Favorite Song. Alpha ERD/ERS (%) vs. eyes-closed baseline.

*Alpha ERD/ERS pattern (music)*: A strong occipital ERS (O2 + 38.8% and O1 + 26.4%) is compatible with the vivid visual imagery reported by the participant, co-occurring with widespread frontotemporal and parietal ERD (C4–36.1%, T3–36.3%, P4–34.9%). Heart rate was lower during music listening than at baseline, consistent with an autonomic shift toward physiological relaxation.

## Subjective report—murottal

*What I feel when I hear murottal is goosebumps and goosebumps standing because I remember the pleasure the creator has given. My image when I hear it is imagining the Arabic writing of the letter. And spell it out imagining the length of the short harakat and what letters are used. Feels more focused when listening to the murottal.*

Heart rate frequency at baseline was 84 beats per minute and at murotal listening was 78 beats per minute. The corresponding alpha ERD/ERS topography is shown in Figure 6.

**Figure 6:**
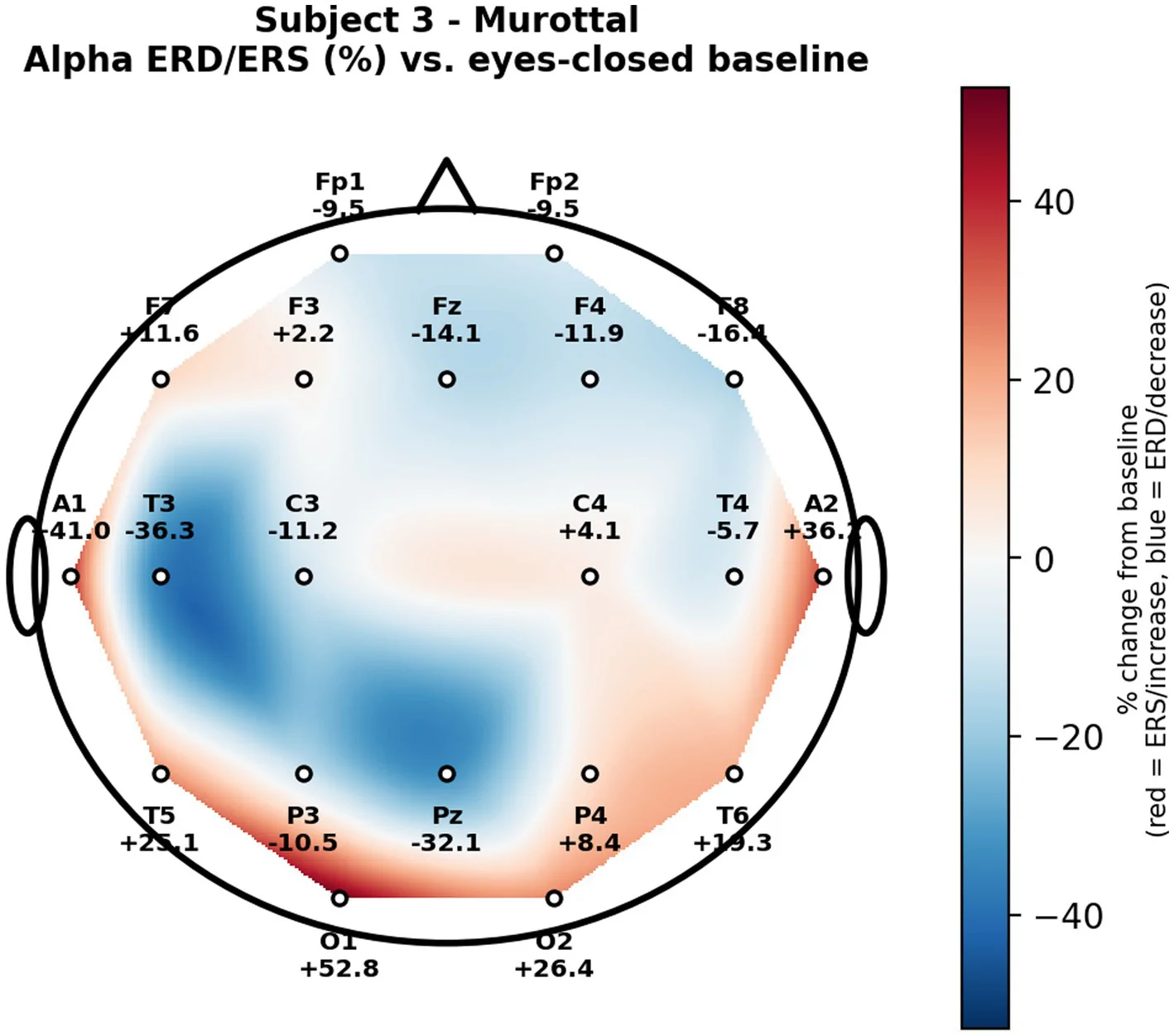
Subject 3 - Murottal. Alpha ERD/ERS (%) vs. eyes-closed baseline.

*Alpha ERD/ERS pattern (murottal)*: Marked occipital ERS (O1 + 52.8%) is compatible with the visual imagery described; ERS in the auricular/temporal regions (A1 + 41.0%, A2 + 36.2%) is compatible with the calm, focused state reported. A broad centroparietal ERD (electrode Pz − 32.1%, C3–11.2%, T3–36.3%) accompanied these increases. The heart rate during Qur’anic murottal listening remained attenuated relative to baseline, consistent with a sustained state of autonomic relaxation.

## Subject 4 (a, 19 y)

### Subjective report—favorite song

The song listened to was “About You” by The 1975, with a beat range of 95–97 bpm.

*When listening to songs, what comes to my mind is the situation on trains and stations, where it is a place where people separate to go overseas, where they start their journey, or as a place to travel to their hometown. At that time, I actually did not have a favorite song; I just thought of it at that time, I happened to be traveling a lot using the train.*

#### Alpha ERD/ERS findings—favorite song

The heart rate at baseline was 60 beats per minute and during music listening was 60 beats per minute. The corresponding alpha ERD/ERS topography is shown in Figure 7.

**Figure 7:**
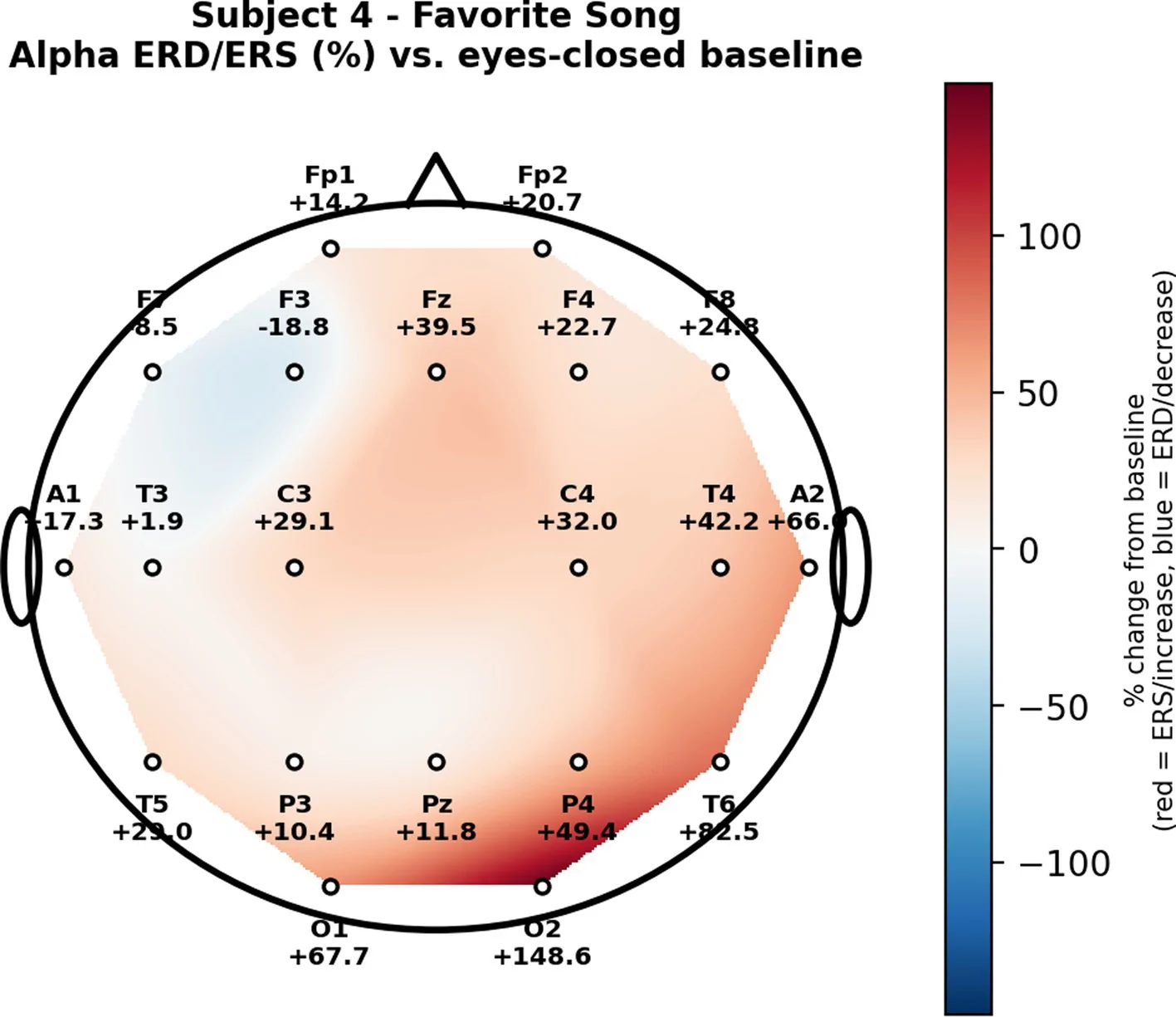
Subject 4 - Favorite Song. Alpha ERD/ERS (%) vs. eyes-closed baseline.

*Alpha ERD/ERS pattern (music listening)*: A marked, right-lateralized occipital ERS (electrode O2 + 148.6%) is compatible with the reported visual/autobiographical imagery described; ERS in the right temporoparietal (T6 + 82.5%) and right auricular (A2 + 66.1%), and midline frontal (Fz + 39.5%) regions is compatible with the reported states of tranquility and sustained focused attention, respectively.

## Subjective report—murottal

*During the examination, the first thing I felt was tension. Because it was my first experience doing and seeing the process firsthand. During the examination, I tried to relax, I tried not to think about anything so as not to get tense. Then, when listening to the murottal, I initially felt tense, because the murottal that was playing was not what I was used to listening to. However, over time I felt relaxed.*

### Alpha ERD/ERS findings—murottal

Heart rate at baseline was 60 beats per minute and 66 beats per minute when listening to murottal. The corresponding alpha ERD/ERS topography is shown in Figure 8.

**Figure 8:**
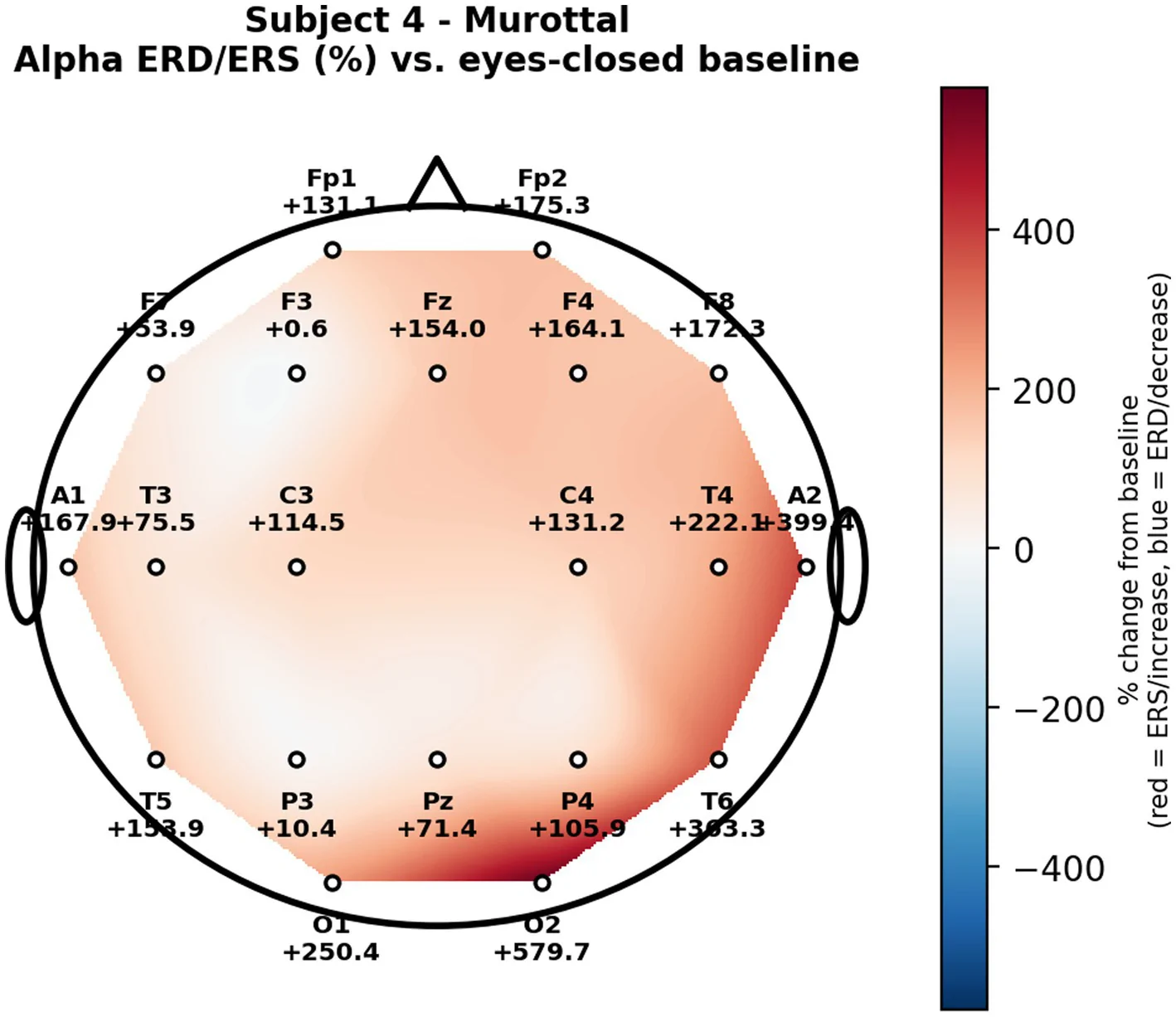
Subject 4 - Murottal. Alpha ERD/ERS (%) vs. eyes-closed baseline.

*Alpha ERD/ERS pattern (Qur’anic murottal listening)*: This recording shows the largest pronounced ERS values observed across the entire series (peaking at electrode O2 + 579.7%, A2 + 399.4%, T6 + 363.3%), a marked and disproportionate relative power increase across nearly every electrode site. Given this participant’s explicit report of situational apprehension, unfamiliarity with the experimental setting, and lack of prior exposure to the reciter, this diffuse, extreme ERS pattern is interpreted as reflecting effortful compensatory concentration and possible muscle-tension artifacts rather than a stimulus-specific murottal auditory neural response—a clear illustration of the fixed-order/context-effect limitations discussed below. The heart rate increased slightly relative to baseline, which is directionally consistent with the participant’s self-reported initial tension.

## Discussion

Four descriptive patterns recurred across the four cases: (1) increases in middle temporal/auricular alpha ERD/ERS plausibly related to task and setting adaptation (a “laboratory effect”), most pronounced in Participant 4; (2) increases in prefrontal alpha ERD/ERS plausibly related to effortful attention toward stimulus content; (3) increases in occipital alpha ERD/ERS plausibly related to visual imagery, present in Participants 1, 3, and 4; and (4) increases in middle-temporal-to-central alpha ERD/ERS co-occurring with subjective reports of calmness and relaxation, most clearly in Participants 1 and 2. These are within-subject, pattern-level co-occurrences rather than statistically validated associations, given the sample size (*N* = 4) and the absence of a counterbalanced control condition or inferential statistical testing.

Listening to music is a highly individual experience, with response differences attributable to both sex and musical expertise (Toiviainen and Alluri, 2023). Music listening can influence stimulus–response timing in women, although it may also impair certain cognitive performance measures in both sexes (Feizpour et al., 2020). Traditional Indonesian karawitan music, like Mozart’s piano sonatas, has been reported to increase frontal lobe activity (Wardani et al., 2023). A 2025 study that explicitly used alpha-ERD as an outcome measure found lower alpha-ERD following music listening than after passive rest, interpreted as a marker of restored attentional capacity (Wang et al., 2025), a finding broadly compatible with the relaxation-associated ERD/ERS patterns observed here in Participants 1–3 in the present study.

Binaural beat therapy, using differing sound frequencies between the right and left ears, has similarly been associated with alpha-wave induction in specific brain regions in several studies (Choi et al., 2022; Yi et al., 2022). The calming, parasympathetically mediated mechanism proposed here is broadly consistent with clinical findings at Universitas Muhammadiyah Surakarta, where a literature review of music therapy in intensive care unit patients similarly reported favorable calming effects on physiological and psychological stress indices (Muzaki and Hudiyawati, 2020), while pre-treatment music listening measurably reduced dental procedure anxiety alongside a reduction in systolic blood pressure (Kurniawati, 2019). Taken together with the qEEG-Quran literature discussed above (Majidi and Rajabi-Tavakkol, 2025) these findings support alpha ERD/ERS as a plausible, if still preliminary, neurophysiological correlate of the relaxation and internally generated mental imagery reported by our participants, while underscoring the need for adequately powered, randomized, and counterbalanced designs before causal or generalizable claims can be established.

This case series has both strengths and limitations that directly inform the interpretation of our findings. Its principal strength lies in the integration of simultaneous subjective (first-person phenomenological reports), physiological (heart rate), and quantitative EEG measurements within the same experimental session, using a Qur’anic murottal stimulus standardized across participants and a personally meaningful comparison stimulus (favorite song), which together allow rich within-subject description. Its principal limitations are, first, the small sample (*N* = 4), restricted to healthy 19-year-old female university students, which inherently limits the generalizability of these observations across broader age cohorts, sex, and clinical status; second, the fixed, non-counterbalanced stimulus order, such that order, fatigue, and habituation effects cannot be fully separated from stimulus-specific effects—most apparent in Participant 4, whose murottal recording shows an extreme, diffuse ERS pattern alongside explicitly reported situational tension; third, the absence of inferential statistics, such that the co-occurrences described above remain observational; and fourth, the fact that alpha ERD/ERS is a block-averaged relative power change that must not be conflated with the classical event-related potential. As this is a single-session experimental design without a clinical intervention, no follow-up or outcomes trajectory in the clinical sense applies, consistent with the descriptive, non-clinical nature of the study. All four participants’ first-person accounts (Serial Report, above) are treated as primary phenomenological data alongside the qEEG measures, in keeping with the study’s phenomenological framing. Fifth, heart rate was originally derived from a 10-s sampling window read directly off the clinical strip, a method susceptible to transient measurement and transcription error; the baseline and listening heart-rate values for Participant 1 originally reported (162 and 144 bpm) were physiologically implausible for a relaxed resting state and have been corrected following calibrated visual counting of QRS complexes on the original raw ECG trace (using the instrument’s own elapsed-time stamps to confirm a 6-s duration per printed page at 30 mm/s paper speed; Supporting File 2). The corrected values (approximately 95–120 bpm across conditions) remain modestly elevated relative to typical resting heart rate in healthy young adults, which may reflect genuine situational anxiety related to the unfamiliar recording setting, consistent with the tension explicitly reported by Participant 4 elsewhere in this case series, rather than a measurement artifact alone. Because this corrected estimate was obtained by manual counting from a scanned printout rather than a software-computed value, it should be treated as an improved approximation rather than an exact instrument readout. Sixth, artifact handling relied on visual inspection of the concurrent EEG and ECG channels to exclude contaminated epochs, rather than a formal automated pipeline such as Independent Component Analysis; the extreme, diffuse alpha ERS values observed in Participant 4’s murottal recording (Section 3.4) are considered most consistent with residual muscle tension or movement artifact under this visual inspection approach, and we flag this as a specific direction for methodological improvement in future work.

## Conclusion

The conscious experience of listening to self-selected favorite music and Qur’anic murottal appears, in this exploratory case series, to be accompanied by highly individualized alpha ERD/ERS topographies that broadly co-occur with participants’ self-reported phenomenological and emotional states. Furthermore, Participant 4’s murottal recording illustrates how situational anxiety and fixed stimulus presentation order can produce disproportionately large spectral power shifts in ERD/ERS values, underscoring the importance of counterbalancing and automated artifact decomposition in future work. Given the descriptive, non-randomized, small-sample design, these findings should be regarded as preliminary and hypothesis-generating. Larger, statistically powered, counterbalanced studies incorporating inferential modeling are needed to determine whether the topographic patterns observed here reflect reliable, generalizable neural correlates of auditory and spiritual neuromodulation.

## Data Availability

The original contributions presented in the study are included in the article/Supplementary material, further inquiries can be directed to the corresponding author/s.
