# Supplementary material for: Subjective experience of listening to favorite songs and Murottal Al-Qur’an in young adult women: a quantitative EEG case series
Source: Front Behav Neurosci. 2026 Sep 24;20:1903142. doi: 10.3389/fnbeh.2026.1903142 (PMC13646118; doi:10.3389/fnbeh.2026.1903142)

KOMISI ETIK PENELITIAN KESEHATAN (KEPK)
Health Research Ethics Committee
FAKULTAS KEDOKTERAN
Universitas Muhammadiyah Surakarta
Faculty of Medicine Universitas Muhammadiyah Surakarta
Komplek kampus 4 UMS Gonilan Kartasura, Telp.(0271)716844, Fax.(0271)724883 Surakarta 57102, email:kepk@ums.ac.id

ETHICAL CLEARANCE LETTER
Surat Kelaiakan Etik
No. 1403/B.2/KEPK-FKUMS/VIII/2023

Komisi Etik Penelitian Kesehatan (KEPK) FK UMS, setelah menelaah rancangan penelitian yang diusulkan menyatakan bahwa:
Health Research Ethics Committee Faculty of medicine of Universitas Muhammadiyah Surakarta, after reviewing the research design, state that:

Penelitian dengan judul:
The research proposal with topic:

Subjective Experience of Listening to Favorite Songs and Murottal Al-Qur'an in Young Adult Women: A Quantitative EEG Case Series

Peneliti:
The researcher:

Nama/ *Name* : **Yusuf Alam Romadhon**

Alamat/ *Address* : Jl Rajawali VII Candibaru Gonilan Kartasura Sukoharjo Jawa Tengah

Institusi/ *Institution* : FAKULTAS KEDOKTERAN UNIVERSITAS MUHAMMADIYAH SURAKARTA

Telah memenuhi deklarasi Helsinki 1975 dan Pedoman nasional etik penelitian kesehatan Departemen Kesehatan RI 2004
Has met the declaration of Helsinki 1975 and national health research ethics Department of Health of the Republic of Indonesia in 2004

dan dinyatakan lolos etik
and ethically approve

Surakarta,
Ketua/*Chairman*,

Prof. Dr. dr. EM. Sutrisna, M,Kes.

Explanatory Cover Sheet

Supporting File 2 (Raw Quantitative EEG Instrument Report (Subject 1))

Manuscript ID 1903142 "Subjective Experience of Listening to Favorite Songs and Murottal Al-Qur'an in Young Adult Women: A Quantitative EEG Case Series"

Purpose of this document

This cover sheet explains how the attached 16-page raw instrument printout (Applied NeuroScience, Inc. qEEG report, EEG ID: Clinical_EEG) relates to the findings reported in the manuscript. The printout documents the original recording session for Subject 1 (K), one of the four participants in the case series, and is provided to the Editor and reviewers as primary-source evidence supporting the manuscript's methodological description and results.

Subject identity and session metadata (confirmed from the printout)

Field	Value on printout	Corresponding manuscript location
Subject name / ID	Khairunnisa, Kana / Subject ID 0105629	Serial Report, Subject 1 (K, 19 years)
Date of birth / age at test	09/11/2004; age 18.79 at test	Consistent with "19 years" for all four participants
Date and time of test	27/06/2023, session starting 08:48:37	Confirms data collection pre-dates manuscript submission
Recording montage / reference	Montage: Default; ipsilateral-ear reference	Observation Method — 20 Ag/AgCl electrodes, 10-20 system
Filter settings	High Cut 35 Hz; Low Cut 0.53 Hz; Notch On; 7 µV/mm; 30 mm/sec	Observation Method — updated to match this source exactly
Analysis software	Applied NeuroScience, Inc. (© 2001–2018), FFT Relative Power module	Observation Method — cited per Reviewer 2, Point 5

Page-by-page contents

Pages	Content	Label	Manuscript section
1	Subject Information cover sheet	—	Confirms Subject 1 identity/age
2–3	FFT Relative Power tables and topographic band maps	"1. eyes close"	Baseline condition preceding favorite-song block
4–5	Raw 20-channel EEG + ECG trace, continuous strip chart	"Eyes Closed"	Source of baseline heart rate and alpha power (B)
6–9	FFT tables, band maps, raw trace for murottal condition	"2. Murotal"	Serial Report, murottal subjective report and HR
10–16	FFT tables, band maps, raw trace for second baseline	"Eyes Closed" (2nd)	Second, independent baseline before familiar music

How this supports the manuscript

1. Confirms that continuous EEG and ECG monitoring was genuinely performed throughout the session, addressing Reviewer 2's request for an explicit account of artifact monitoring during recording.

2. Provides the source filter settings, montage, reference scheme, and analysis software now stated explicitly in the revised Observation Method, addressing Reviewer 2's request for acquisition and analysis software details.

3. Documents that a separate, independent eyes-closed baseline was recorded before each listening condition, supporting the manuscript's clarification that the two baseline heart-rate/alpha-power values reported per participant are not a single shared measurement.

4. The continuous ECG channel on the raw trace is the source of the corrected heart-rate values reported for Subject 1. The original values (162/144 bpm) were physiologically implausible; the corrected values (approximately 95–120 bpm) were derived by calibrated visual counting of QRS complexes on this raw trace, using the instrument's own elapsed-time stamps to confirm timing (see Discussion, limitations paragraph, for full methodology and caveats).

Note on scope

This printout covers Subject 1 only, as this was the file available at the time of this submission. The corresponding raw printouts for Subjects 2–4 follow the same acquisition protocol, montage, and software, and can be supplied on request.

Appendix — Heart-Rate Correction Evidence

The figures below are cropped, enlarged excerpts of the ECG channel from the raw trace pages of the attached printout, provided as direct visual evidence for the heart-rate correction described in the manuscript (Discussion, limitations paragraph) and in the response to Reviewer 2, Point 1. Each excerpt spans approximately 3 seconds of continuous recording, calibrated using the instrument's own elapsed-time stamps (one full printed page = 6 seconds at the stated 30 mm/sec paper speed). Distinct QRS complexes (the sharp upward deflections) are visible and were counted manually; the resulting rate is reported as an approximate range rather than an exact value, reflecting the inherent limits of manual counting from a scanned printout.

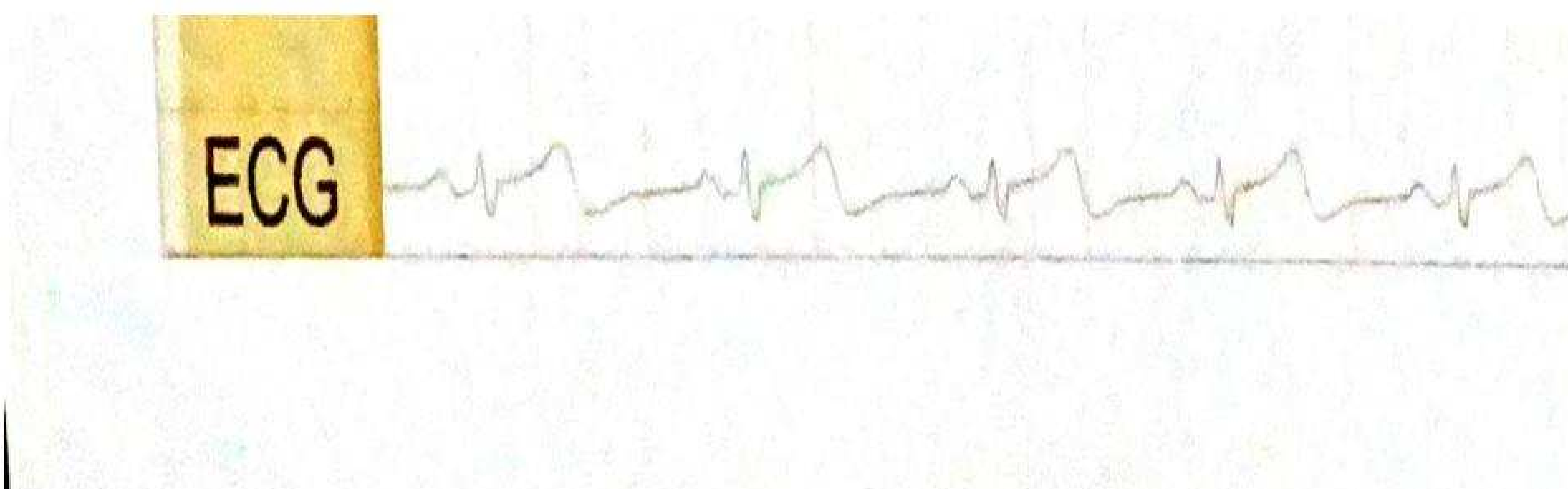

Subject 1 - baseline ECG before favorite song (raw trace, ~3 sec window). Visual QRS count -> approx. 100-115 bpm.

Figure A1. Raw ECG excerpt, baseline immediately preceding the favorite-song condition (printout pages ~4-5).

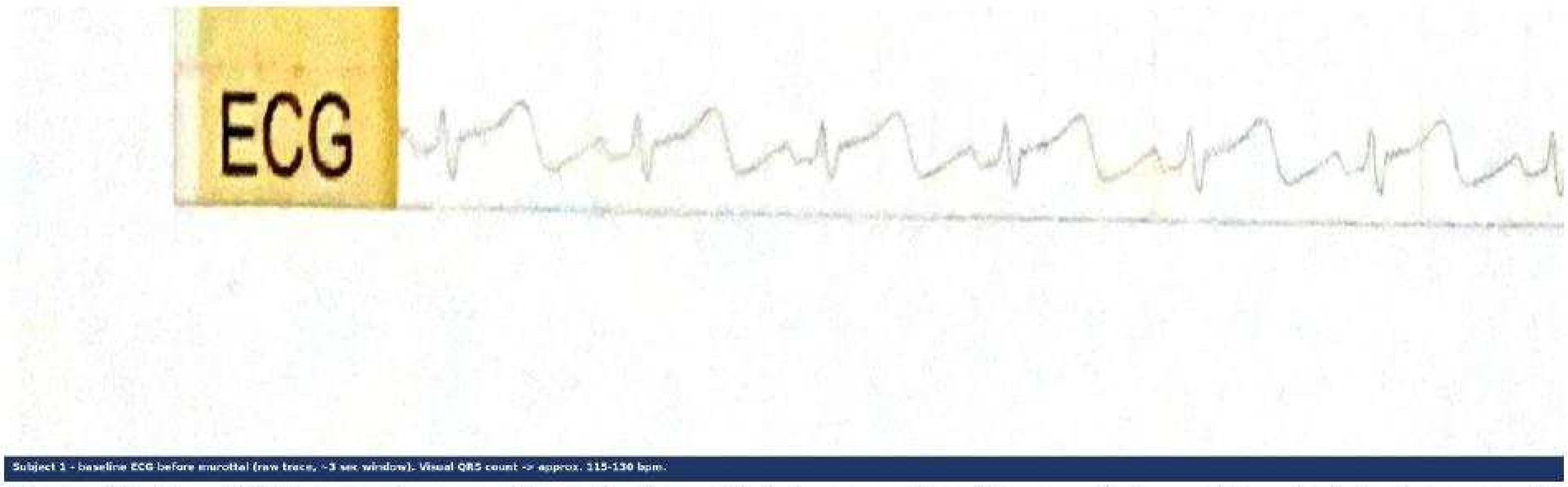

Figure A2. Raw ECG excerpt, second baseline immediately preceding the murottal condition (printout pages ~15-16).

Both excerpts show a regular rhythm with clearly resolvable individual beats at roughly 100-130 bpm, consistent with the corrected values now reported in the manuscript (approximately 95-120 bpm across conditions) and clearly inconsistent with the originally reported 162 bpm. We present these excerpts transparently, without claiming instrument-grade precision, so that the Editor and reviewers may verify the correction independently.

Appendix B - Cross-Check: Raw FFT Alpha Power vs. Manuscript ERD/ERS Values (Subject 1, Murottal)

To independently verify the manuscript's reported alpha ERD/ERS topography, we cross-checked it against the ALPHA column of the instrument's own automated FFT Relative Power report, comparing the baseline ('1. eyes close') and murottal ('2. Murotal') pages for Subject 1, using the manuscript's own formula: ERD/ERS (%) = (E - B)/B x 100.

FFT Relative Power (%)

Intrahemispheric: LEFT

<- column used for ERD/ERS calc

	DELTA	THETA	ALPHA	BETA	HIGH	BETA 1	BETA 2	BETA 3
FP1 - Cz	48.93	15.12	18.45	6.11	1.12	2.42	1.23	2.47
F3 - Cz	25.30	17.17	29.38	8.20	1.04	3.28	1.60	3.32
C3 - Cz	17.50	17.10	44.29	10.02	0.88	3.95	1.82	4.25
P3 - Cz	11.65	11.56	63.34	7.60	0.51	2.92	1.23	3.45
O1 - Cz	8.85	8.88	63.62	6.15	0.34	2.52	1.30	2.33
F7 - Cz	28.61	14.58	23.64	6.81	0.89	2.68	1.34	2.80
T3 - Cz	17.13	15.44	38.61	9.45	1.23	3.30	1.96	4.19
T5 - Cz	13.24	13.92	55.80	8.05	0.58	3.08	1.62	3.35
FT9 - Cz	23.07	14.06	29.23	7.15	0.76	2.80	1.54	2.81
A1 - Cz	19.36	14.87	41.59	8.87	0.88	3.38	1.98	3.51

Intrahemispheric: RIGHT

<- column used

	DELTA	THETA	ALPHA	BETA	HIGH	BETA 1	BETA 2	BETA 3
FP2 - Cz	52.64	15.19	17.59	5.63	0.84	2.08	1.16	2.39
F4 - Cz	33.74	17.77	32.40	9.40	1.13	3.36	2.03	4.01
C4 - Cz	18.35	15.19	52.27	11.58	0.83	4.36	2.32	4.90
P4 - Cz	10.93	9.67	61.95	8.24	0.47	3.45	1.76	3.02
O2 - Cz	8.37	8.50	74.42	7.69	0.37	3.42	2.03	2.24
F8 - Cz	36.56	18.88	30.78	9.18	0.97	3.39	2.08	3.71
T4 - Cz	22.76	17.74	45.55	11.53	0.90	4.24	2.53	4.76
T6 - Cz	13.91	12.89	62.11	9.81	0.55	4.05	2.37	3.39
FT10 - Cz	32.97	18.77	36.28	9.36	0.91	3.45	2.30	3.61
A2 - Cz	22.22	16.96	48.10	10.01	0.84	3.70	2.60	3.71

Intrahemispheric: CENTER

<- column used

	DELTA	THETA	ALPHA	BETA	HIGH	BETA 1	BETA 2	BETA 3
Fz - Cz	33.10	16.25	27.31	6.74	0.97	2.72	1.30	2.72
Pz - Cz	12.85	10.08	69.36	6.69	0.43	2.82	1.19	2.69

ALPHA column (red boxes) = raw alpha power (%) per electrode,
condition: Murottal listening (Subject 1). Used as 'E' in (E-B)/B x 100%.
Reference: Cz (this table) -- differs from ipsi-ear reference used for the manuscript's raw trace.

Figure B1. Raw FFT Relative Power table, murottal condition, with the ALPHA column (used as E) highlighted.

Result: for 4 of 22 electrodes (Pz, C4, P4, O2) the value computed from this raw table closely matches the manuscript's reported figure (within a few percentage points, same direction); for 6 electrodes the direction matches but the magnitude differs; for the remaining electrodes -- concentrated at frontal sites (Fp1, Fp2, F3, F4, F7, F8, Fz) and the ear/auricular and temporal channels (A1, A2, T3, T4, T5, T6) -- the computed value differs substantially and sometimes in direction from the manuscript's reported figure.

We believe the most likely explanation is a reference-montage difference rather than fabricated data: this automated FFT report is referenced to Cz (labelled 'Fp1-Cz', 'F3-Cz', etc.), whereas the continuous raw 20-channel trace elsewhere in this printout is explicitly recorded with an ipsilateral-ear reference ('Montage: ipsi ear ref'), which is also the reference scheme described in the manuscript's Observation Method. Because a change of reference electrode mathematically alters the derived potential at every other channel, the two are not expected to match electrode-for-electrode. The strongest matches (central and posterior sites: C4, P4, Pz, O2) are consistent with this explanation, as these sites are least affected by the choice between a Cz and an ear reference; frontal and temporal sites, which are most sensitive to reference choice (and to eyelid/muscle artifact), show the largest discrepancies.

We flag this transparently rather than presenting it as full independent verification: the raw FFT table confirms that alpha power was recorded and generally increased from baseline to murottal at most electrodes, consistent with the manuscript's qualitative conclusions, but it does not, on its own, reproduce the manuscript's electrode-by-electrode figures exactly. We recommend the corresponding author confirm which montage/reference was used to derive the published topoplots, and, if it was the ipsi-ear reference, supply the corresponding ipsi-ear-referenced alpha power table (rather than this Cz-referenced FFT summary) as the definitive source data in any future revision.

Electrode	Baseline alpha (%)	Murottal alpha (%)	Computed (E-B)/B x100%	Manuscript-reported %	Verdict
Fp1	14.89	18.45	+23.91	-27.45	Mismatch
F7	14.88	23.64	+58.87	-10.18	Mismatch
F3	21.78	29.38	+34.89	-8.33	Mismatch
Fz	27.12	27.31	+0.70	-17.79	Mismatch
A1	34.05	41.59	+22.14	-1.66	Mismatch
T3	29.93	38.61	+29.00	-4.92	Mismatch
C3	35.77	44.29	+23.82	+3.46	Partial
T5	47.45	55.80	+17.60	-0.69	Mismatch
P3	59.98	63.34	+5.60	-2.15	Mismatch
O1	57.70	63.62	+10.26	+1.94	Partial
Fp2	12.19	17.59	+44.30	-17.80	Mismatch
F8	17.93	30.78	+71.67	-1.19	Mismatch
F4	21.56	32.40	+50.28	+5.19	Partial
Pz	74.09	69.36	-6.38	-5.61	Match
C4	38.47	52.27	+35.87	+35.38	Match
P4	56.31	61.95	+10.02	+4.50	Match
T4	34.30	45.55	+32.80	+16.29	Partial
T6	55.56	62.11	+11.79	+3.31	Partial
A2	39.04	48.10	+23.21	-3.65	Mismatch
O2	71.56	74.42	+4.00	+0.96	Match

Table B1. Electrode-by-electrode cross-check, Subject 1 murottal condition. Green = match, yellow = same direction/different magnitude, red = mismatch.

① eyes close (1)

FFT Relative Power (%)

Intrahemispheric: LEFT

	DELTA	THETA	ALPHA	BETA	HIGH	BETA 1	BETA 2	BETA 3
FP1 - Cz	58.06	12.79	14.89	5.04	0.88	1.72	1.26	2.06
F3 - Cz	37.77	17.19	21.78	6.72	1.02	2.24	1.66	2.82
C3 - Cz	25.22	18.81	35.77	9.20	1.15	3.15	2.15	3.90
P3 - Cz	12.38	13.96	59.98	7.71	0.57	2.85	1.59	3.27
O1 - Cz	10.37	11.96	57.70	6.68	0.39	2.47	1.96	2.25
F7 - Cz	47.54	14.30	14.88	4.74	0.69	1.64	1.17	1.93
T3 - Cz	23.14	16.88	29.93	9.55	1.94	2.51	2.05	5.00
T5 - Cz	17.23	17.30	47.45	8.30	0.77	2.94	2.21	3.14
FT9 - Cz	31.19	15.71	21.97	6.43	0.93	2.32	1.58	2.53
A1 - Cz	26.19	17.49	34.05	7.20	0.66	2.67	2.01	2.52

Intrahemispheric: RIGHT

	DELTA	THETA	ALPHA	BETA	HIGH	BETA 1	BETA 2	BETA 3
FP2 - Cz	66.54	11.24	12.19	3.80	0.63	1.41	0.94	1.45
F4 - Cz	50.61	15.71	21.56	7.03	0.98	2.39	1.85	2.78
C4 - Cz	32.85	16.88	38.47	9.22	0.94	3.01	2.02	4.18
P4 - Cz	14.14	12.37	56.31	7.39	0.57	2.64	1.79	2.96
O2 - Cz	10.51	10.20	71.56	6.75	0.40	2.85	1.90	2.00
F8 - Cz	59.03	14.63	17.93	5.38	0.67	2.02	1.27	2.09
T4 - Cz	35.57	17.88	34.30	9.33	1.16	3.17	2.20	3.96
T6 - Cz	19.05	15.36	55.56	8.69	0.65	3.28	2.28	3.12
FT10 - Cz	49.19	16.60	24.47	7.05	0.85	2.82	1.64	2.60
A2 - Cz	30.69	18.69	39.04	8.71	0.82	3.41	2.30	3.01

Intrahemispheric: CENTER

	DELTA	THETA	ALPHA	BETA	HIGH	BETA 1	BETA 2	BETA 3
Fz - Cz	33.93	15.95	27.12	7.07	0.99	2.53	1.84	2.70
Pz - Cz	10.01	9.11	74.09	5.87	0.46	2.08	1.38	2.40

© 2001-2018, Applied NeuroScience, Inc.

FFT Relative Power (%)

Warning: Absolute power must be consulted to interpret relative power.

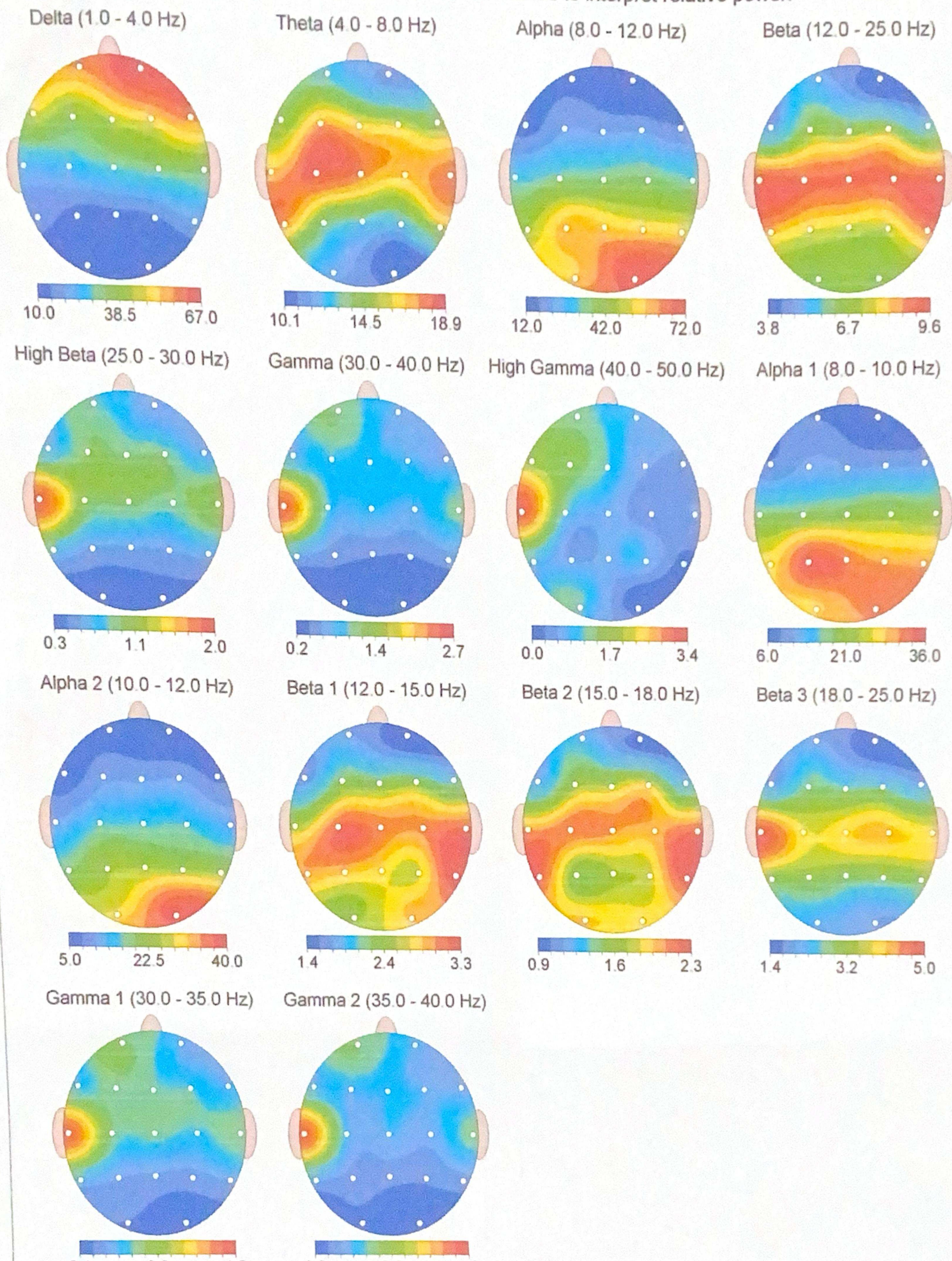

© 2001-2018, Applied NeuroScience, Inc.

KHAIRUNNISA, KANA KHAIRUNNISA 2023-06-27 08-48-37, Page# 12, 09:22:01, ET: 00:01:54, Montage: ipsi ear ref rsjd, Paper Speed: 30 mm/sec
High Cut: 35 Hz, Low Cut: 0.53 Hz, Notch: On, Sensitivity: 7 µV/mm

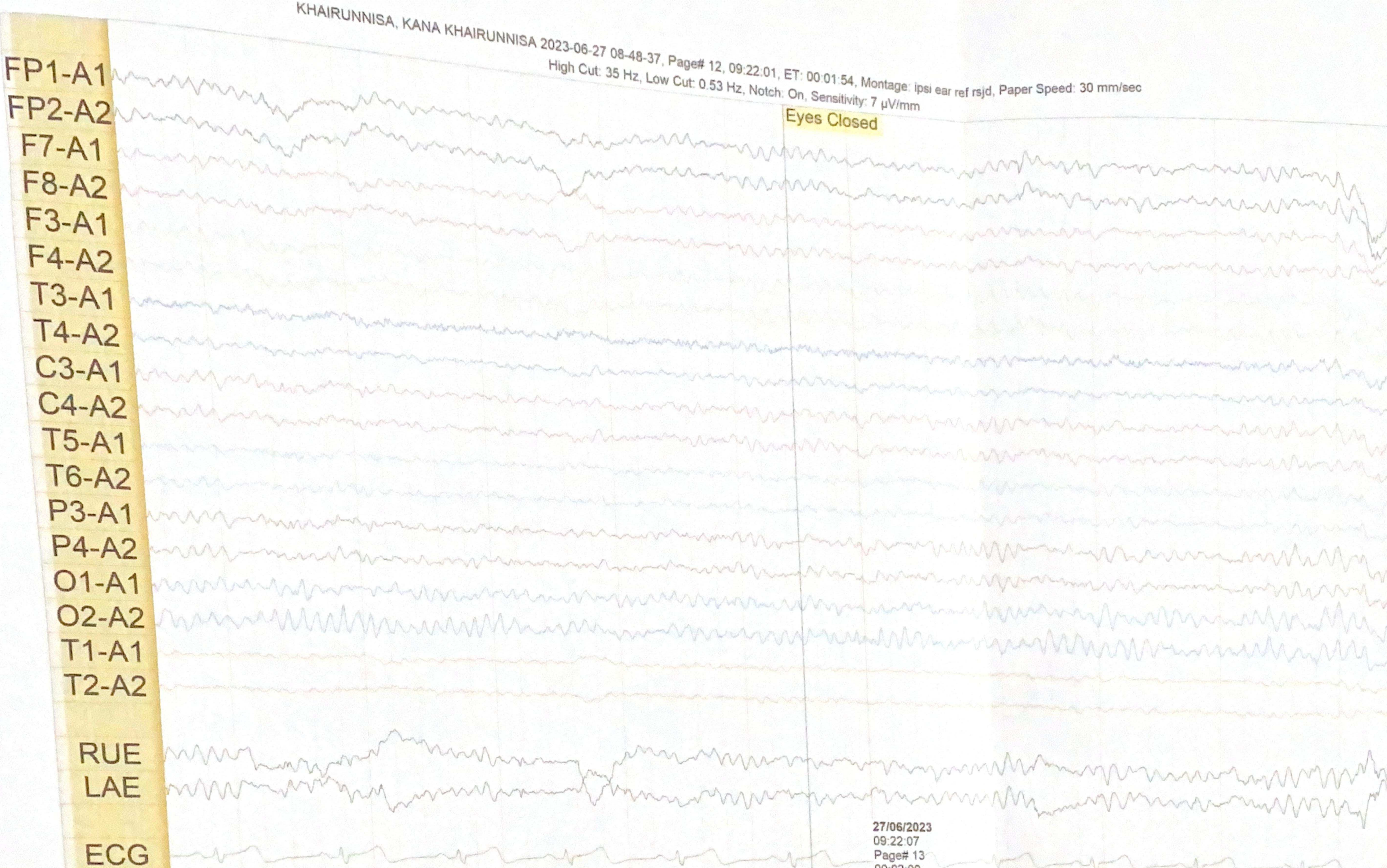

Subject Information

Name:	KHAIRUNNISA_KANA
Subject ID:	0105629
Date of Birth:	09/11/2004
Age:	18.79
Gender:	F
Handedness:	
EEG ID:	Clinical_EEG
Date of Test:	06/27/2023
Time of Test:	09:20:06
Technician:	Admin
Eyes Condition:	Eyes Closed
Clinician:	
Medication:	
Comments:	

© 2001-2018, Applied NeuroScience, Inc.

2. Murotal

FFT Relative Power (%)

Intrahemispheric: LEFT

	DELTA	THETA	ALPHA	BETA	HIGH	BETA 1	BETA 2	BETA 3
FP1 - Cz	48.93	15.12	18.45	6.11	1.12	2.42	1.23	2.47
F3 - Cz	25.30	17.17	29.38	8.20	1.04	3.28	1.60	3.32
C3 - Cz	17.50	17.10	44.29	10.02	0.88	3.95	1.82	4.25
P3 - Cz	11.65	11.56	63.34	7.60	0.51	2.92	1.23	3.45
O1 - Cz	8.85	8.88	63.62	6.15	0.34	2.52	1.30	2.33
F7 - Cz	28.61	14.58	23.64	6.81	0.89	2.68	1.34	2.80
T3 - Cz	17.13	15.44	38.61	9.45	1.23	3.30	1.96	4.19
T5 - Cz	13.24	13.92	55.80	8.05	0.58	3.08	1.62	3.35
FT9 - Cz	23.07	14.06	29.23	7.15	0.76	2.80	1.54	2.81
A1 - Cz	19.36	14.87	41.59	8.87	0.88	3.38	1.98	3.51

Intrahemispheric: RIGHT

	DELTA	THETA	ALPHA	BETA	HIGH	BETA 1	BETA 2	BETA 3
FP2 - Cz	52.64	15.19	17.59	5.63	0.84	2.08	1.16	2.39
F4 - Cz	33.74	17.77	32.40	9.40	1.13	3.36	2.03	4.01
C4 - Cz	18.35	15.19	52.27	11.58	0.83	4.36	2.32	4.90
P4 - Cz	10.93	9.67	61.95	8.24	0.47	3.45	1.76	3.02
O2 - Cz	8.37	8.50	74.42	7.69	0.37	3.42	2.03	2.24
F8 - Cz	36.56	18.88	30.78	9.18	0.97	3.39	2.08	3.71
T4 - Cz	22.76	17.74	45.55	11.53	0.90	4.24	2.53	4.76
T6 - Cz	13.91	12.89	62.11	9.81	0.55	4.05	2.37	3.39
FT10 - Cz	32.97	18.77	36.28	9.36	0.91	3.45	2.30	3.61
A2 - Cz	22.22	16.96	48.10	10.01	0.84	3.70	2.60	3.71

Intrahemispheric: CENTER

	DELTA	THETA	ALPHA	BETA	HIGH	BETA 1	BETA 2	BETA 3
Fz - Cz	33.10	16.25	27.31	6.74	0.97	2.72	1.30	2.72
Pz - Cz	12.85	10.08	69.36	6.69	0.43	2.82	1.19	2.69

© 2001-2018, Applied NeuroScience, Inc.

Montage: Default

EEG ID: Clinical_EEG

FFT Relative Power (%)

Warning: Absolute power must be consulted to interpret relative power.

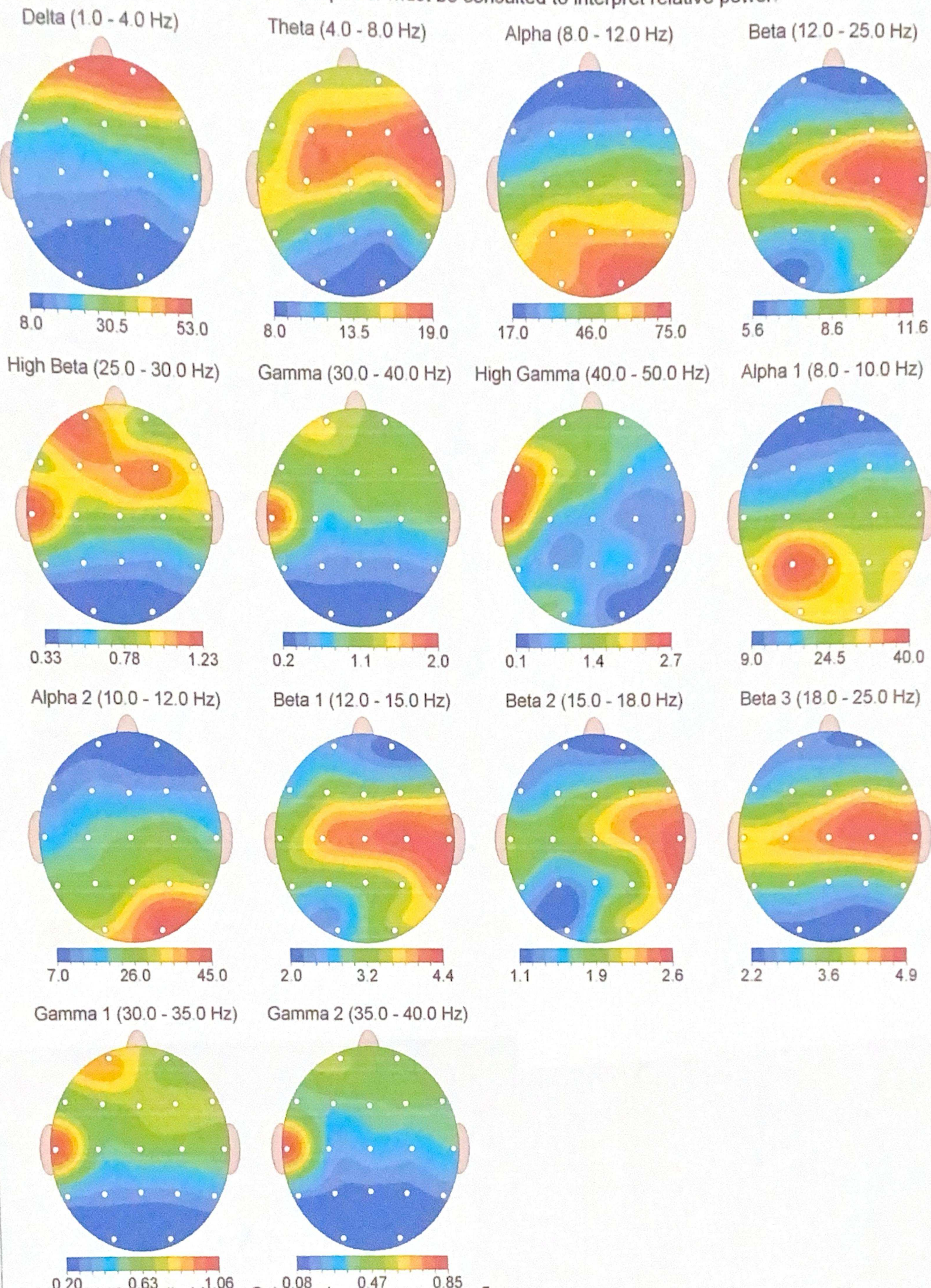

© 2001-2018, Applied NeuroScience, Inc.

KHAIRUNNISA, KANA KHAIRUNNISA 2023-06-27 08-48-37, Page# 40, 09:26:41, ET: 00:06:34, Montage: ipsi ear ref rsjd, Paper Speed: 30 mm/sec
High Cut: 35 Hz, Low Cut: 0.53 Hz, Notch: On, Sensitivity: 7 µV/mm

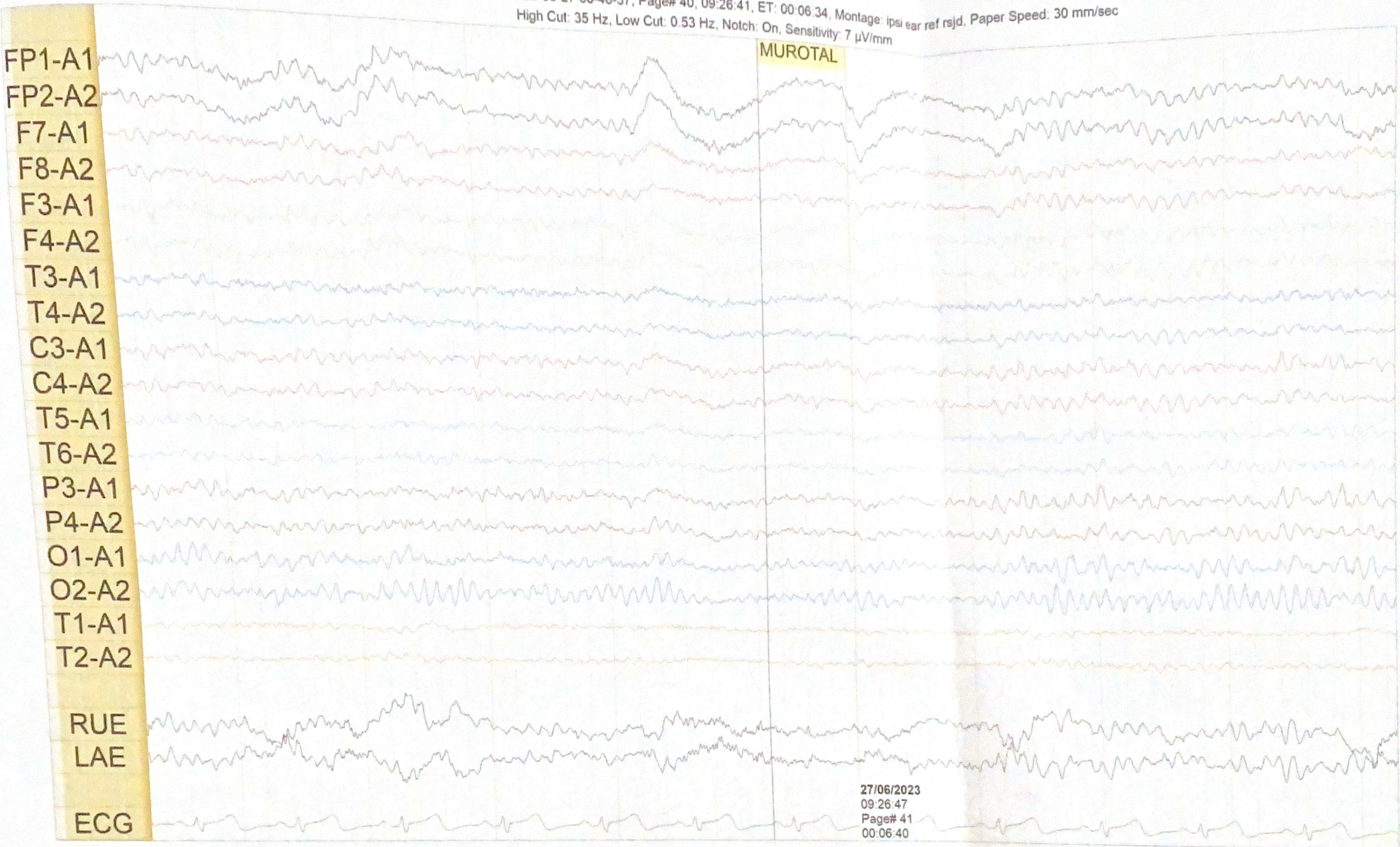

3 Eyes closed (2)

FFT Relative Power (%)

Intrahemispheric: LEFT

	DELTA	THETA	ALPHA	BETA	HIGH	BETA 1	BETA 2	BETA 3
FP1 - Cz	40.00	15.00	25.43	7.19	1.12	2.45	1.37	3.37
F3 - Cz	24.12	14.88	32.05	9.66	1.39	3.11	2.14	4.40
C3 - Cz	17.07	13.59	42.81	12.98	2.21	4.25	2.62	6.11
P3 - Cz	10.74	11.54	64.73	7.56	0.66	2.68	1.53	3.35
O1 - Cz	8.83	10.36	62.41	6.21	0.41	2.24	1.59	2.38
F7 - Cz	26.49	13.75	26.32	7.41	1.08	2.36	1.54	3.50
T3 - Cz	15.85	14.02	40.61	11.32	1.23	2.73	2.75	5.83
T5 - Cz	12.73	14.12	56.19	8.14	0.72	2.60	1.89	3.66
FT9 - Cz	19.10	14.52	31.63	8.11	0.90	2.30	1.88	3.93
A1 - Cz	16.30	15.98	42.29	9.24	0.95	2.82	2.33	4.09

Intrahemispheric: RIGHT

	DELTA	THETA	ALPHA	BETA	HIGH	BETA 1	BETA 2	BETA 3
FP2 - Cz	49.86	12.90	21.40	5.88	1.10	1.76	1.32	2.80
F4 - Cz	27.21	13.97	30.80	11.41	3.10	2.55	2.35	6.51
C4 - Cz	11.43	10.68	38.61	18.90	4.90	3.33	2.58	12.99
P4 - Cz	9.13	10.50	59.28	9.62	1.17	3.08	1.91	4.62
O2 - Cz	7.51	9.45	73.71	7.99	0.42	2.96	2.08	2.96
F8 - Cz	29.35	15.10	31.15	12.37	2.28	2.66	2.84	6.88
T4 - Cz	15.69	14.22	39.17	17.77	3.65	3.64	3.64	10.49
T6 - Cz	12.08	14.10	60.12	11.02	0.79	3.73	2.62	4.67
FT10 - Cz	24.72	18.02	39.68	11.81	1.50	3.03	2.81	5.97
A2 - Cz	16.73	17.77	49.92	11.37	1.04	3.29	3.01	5.07

Intrahemispheric: CENTER

	DELTA	THETA	ALPHA	BETA	HIGH	BETA 1	BETA 2	BETA 3
Fz - Cz	28.25	16.57	33.22	7.19	0.87	2.63	1.43	3.12
Pz - Cz	8.84	10.22	73.48	6.48	0.41	2.38	1.31	2.79

© 2001-2018, Applied NeuroScience, Inc.

Montage: Default EEG ID: Clinical_EEG

FFT Relative Power (%)

Warning: Absolute power must be consulted to interpret relative power.

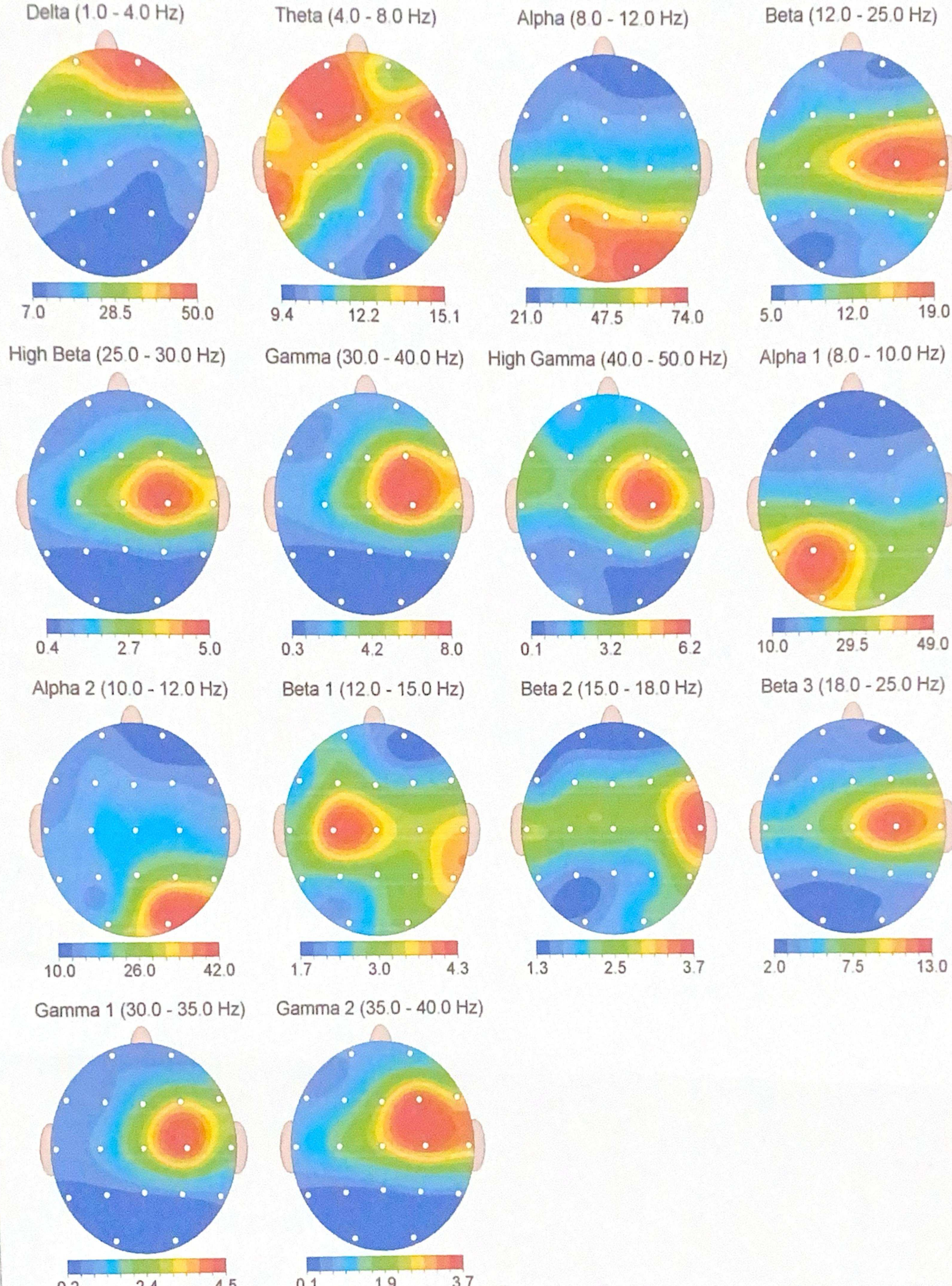

© 2001-2018, Applied NeuroScience, Inc.

KHAIRUNNISA, KANA KHAIRUNNISA 2023-06-27 08-48-37, Page# 60, 09:30:01, ET: 00:09:54, Montage: ipsi ear ref rsjd, Paper Speed: 30 mm/sec
High Cut: 35 Hz, Low Cut: 0.53 Hz, Notch: On, Sensitivity: 7 µV/mm

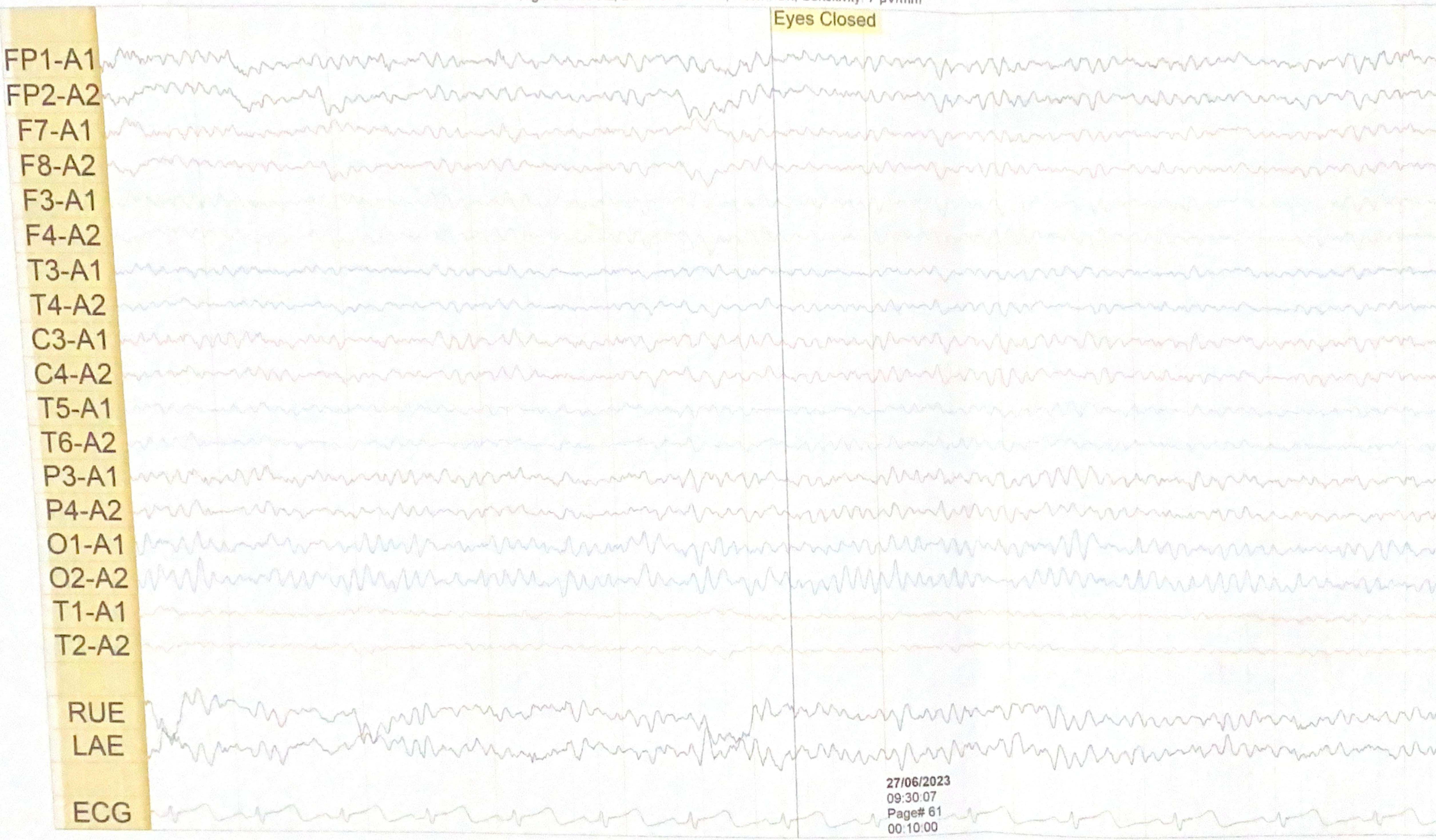

(14) logu

FFT Relative Power (%)

Intrahemispheric: LEFT

	DELTA	THETA	ALPHA	BETA	HIGH	BETA 1	BETA 2	BETA 3
FP1 - Cz	49.74	13.44	21.46	5.24	0.89	1.75	1.16	2.33
F3 - Cz	24.08	17.27	32.90	7.49	1.02	2.36	1.71	3.42
C3 - Cz	16.43	16.34	47.37	9.78	0.87	3.45	1.86	4.47
P3 - Cz	10.67	12.30	63.60	8.28	0.51	2.95	1.40	3.92
O1 - Cz	8.09	10.23	62.83	6.57	0.41	2.41	1.61	2.55
F7 - Cz	22.58	15.39	29.19	6.38	0.90	2.10	1.44	2.85
T3 - Cz	14.96	15.47	44.38	7.92	0.87	2.39	1.89	3.64
T5 - Cz	11.66	14.28	57.81	7.81	0.61	2.56	1.80	3.45
FT9 - Cz	20.06	14.82	34.09	6.52	0.76	2.10	1.54	2.88
A1 - Cz	18.53	15.63	45.29	7.10	0.65	2.28	1.87	2.95

Intrahemispheric: RIGHT

	DELTA	THETA	ALPHA	BETA	HIGH	BETA 1	BETA 2	BETA 3
FP2 - Cz	53.63	13.61	20.22	5.02	0.79	1.67	1.17	2.18
F4 - Cz	29.60	19.39	36.64	8.65	1.01	2.93	1.73	3.98
C4 - Cz	15.19	17.23	54.01	10.97	0.86	3.96	1.84	5.17
P4 - Cz	9.30	11.59	62.06	7.66	0.52	2.84	1.50	3.31
O2 - Cz	7.29	9.92	74.91	6.84	0.37	2.68	1.73	2.42
F8 - Cz	29.51	20.40	37.31	8.41	0.99	2.88	1.84	3.70
T4 - Cz	17.16	19.70	50.69	10.29	0.92	3.64	2.12	4.54
T6 - Cz	11.11	14.92	63.62	8.95	0.63	3.28	2.05	3.61
FT10 - Cz	25.31	19.50	44.49	8.32	0.90	2.79	2.03	3.50
A2 - Cz	16.52	17.45	54.87	8.75	0.79	2.86	2.38	3.52

Intrahemispheric: CENTER

	DELTA	THETA	ALPHA	BETA	HIGH	BETA 1	BETA 2	BETA 3
Fz - Cz	33.26	16.08	30.47	6.06	0.84	2.00	1.27	2.79
Pz - Cz	12.20	13.21	67.97	5.64	0.46	2.29	1.02	2.33

© 2001-2018, Applied NeuroScience, Inc.

EEG ID: Clinical_EEG

FFT Relative Power (%)

Warning: Absolute power must be consulted to interpret relative power.

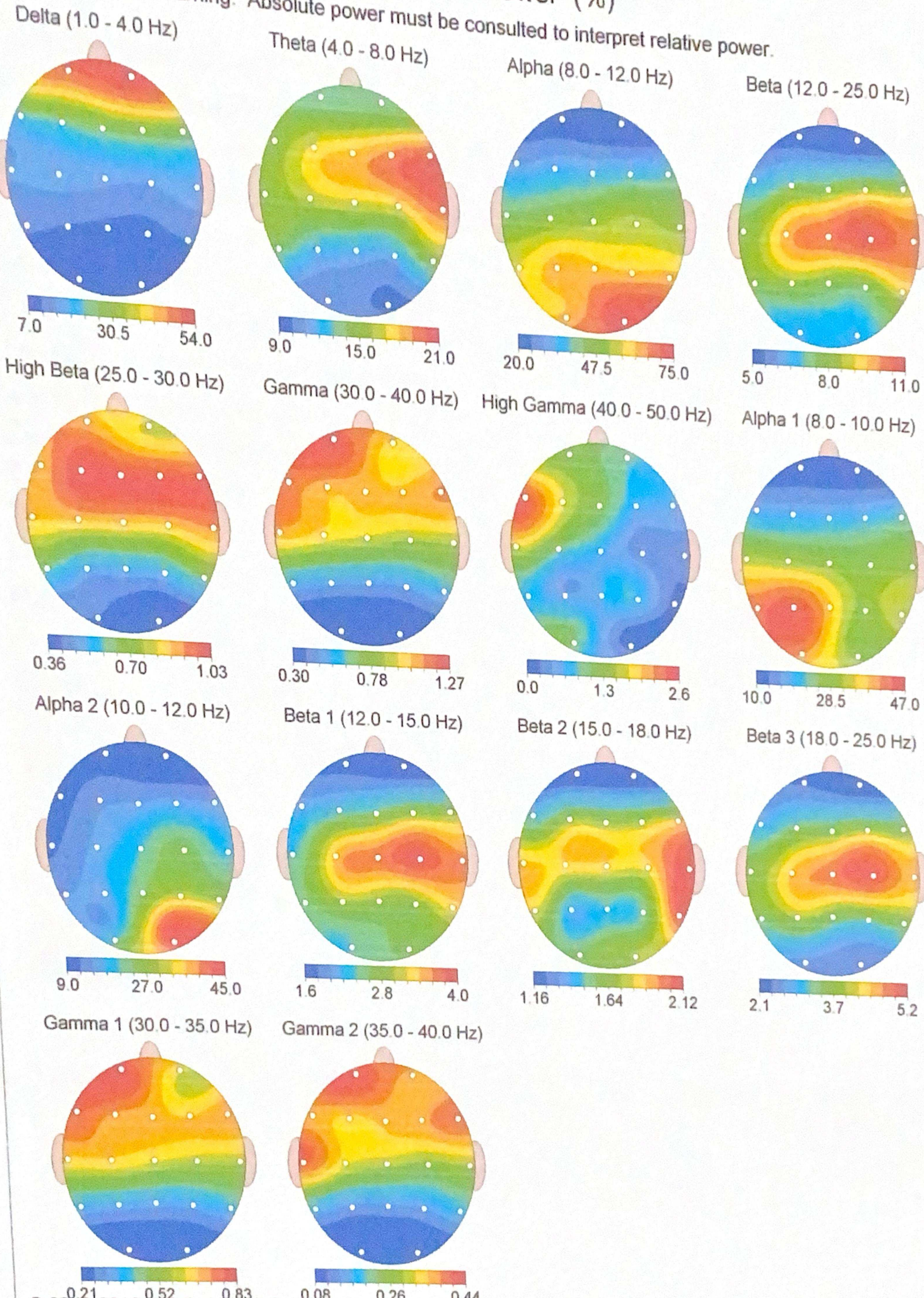

© 2001-2018, Applied NeuroScience, Inc.

KHAIRUNNISA, KANA KHAIRUNNISA 2023-06-27 08-48-37, Page# 86, 09:34:21, ET: 00:14:14, Montage: ipsi ear ref rsjd, Paper Speed: 30 mm/sec
High Cut: 35 Hz, Low Cut: 0.53 Hz, Notch: On, Sensitivity: 7 µV/mm

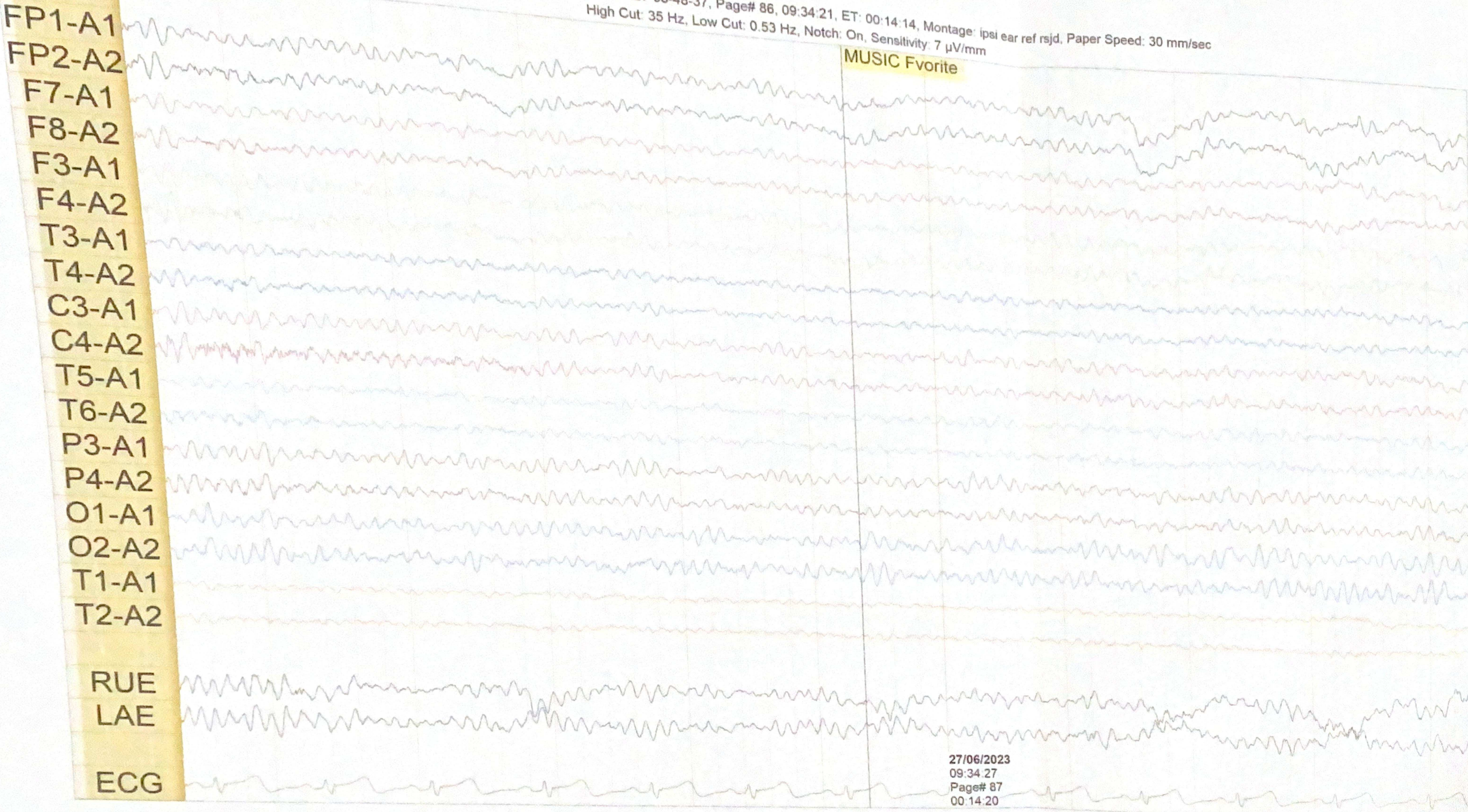

3. Eyes closed (2)

FFT Relative Power (%)

Intrahemispheric: LEFT

	DELTA	THETA	ALPHA	BETA	HIGH	BETA 1	BETA 2	BETA 3
FP1 - Cz	40.00	15.00	25.43	7.19	1.12	2.45	1.37	3.37
F3 - Cz	24.12	14.88	32.05	9.66	1.39	3.11	2.14	4.40
C3 - Cz	17.07	13.59	42.81	12.98	2.21	4.25	2.62	6.11
P3 - Cz	10.74	11.54	64.73	7.56	0.66	2.68	1.53	3.35
O1 - Cz	8.83	10.36	62.41	6.21	0.41	2.24	1.59	2.38
F7 - Cz	26.49	13.75	26.32	7.41	1.08	2.36	1.54	3.50
T3 - Cz	15.85	14.02	40.61	11.32	1.23	2.73	2.75	5.83
T5 - Cz	12.73	14.12	56.19	8.14	0.72	2.60	1.89	3.66
FT9 - Cz	19.10	14.52	31.63	8.11	0.90	2.30	1.88	3.93
A1 - Cz	16.30	15.98	42.29	9.24	0.95	2.82	2.33	4.09

Intrahemispheric: RIGHT

	DELTA	THETA	ALPHA	BETA	HIGH	BETA 1	BETA 2	BETA 3
FP2 - Cz	49.86	12.90	21.40	5.88	1.10	1.76	1.32	2.80
F4 - Cz	27.21	13.97	30.80	11.41	3.10	2.55	2.35	6.51
C4 - Cz	11.43	10.68	38.61	18.90	4.90	3.33	2.58	12.99
P4 - Cz	9.13	10.50	59.28	9.62	1.17	3.08	1.91	4.62
O2 - Cz	7.51	9.45	73.71	7.99	0.42	2.96	2.08	2.96
F8 - Cz	29.35	15.10	31.15	12.37	2.28	2.66	2.84	6.88
T4 - Cz	15.69	14.22	39.17	17.77	3.65	3.64	3.64	10.49
T6 - Cz	12.08	14.10	60.12	11.02	0.79	3.73	2.62	4.67
FT10 - Cz	24.72	18.02	39.68	11.81	1.50	3.03	2.81	5.97
A2 - Cz	16.73	17.77	49.92	11.37	1.04	3.29	3.01	5.07

Intrahemispheric: CENTER

	DELTA	THETA	ALPHA	BETA	HIGH	BETA 1	BETA 2	BETA 3
Fz - Cz	28.25	16.57	33.22	7.19	0.87	2.63	1.43	3.12
Pz - Cz	8.84	10.22	73.48	6.48	0.41	2.38	1.31	2.79

© 2001-2018, Applied NeuroScience, Inc.

FFT Relative Power (%)

Warning: Absolute power must be consulted to interpret relative power.

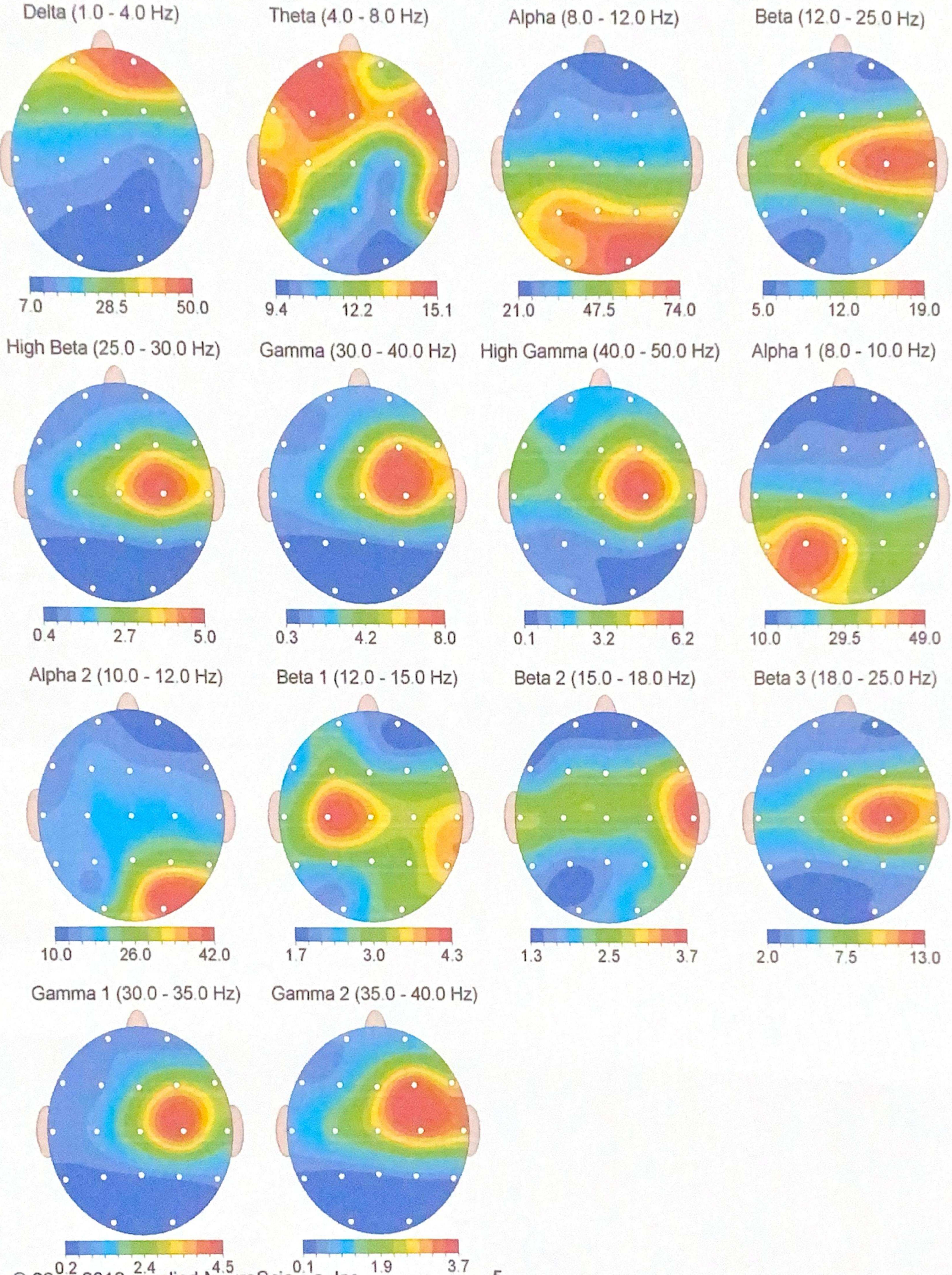

© 2001-2018, Applied NeuroScience, Inc.

KHAIRUNNISA, KANA KHAIRUNNISA 2023-06-27 08-48-37, Page# 60, 09:30:01, ET: 00:09:54, Montage: ipsi ear ref rsjd, Paper Speed: 30 mm/sec
High Cut: 35 Hz, Low Cut: 0.53 Hz, Notch: On, Sensitivity: 7 μV/mm

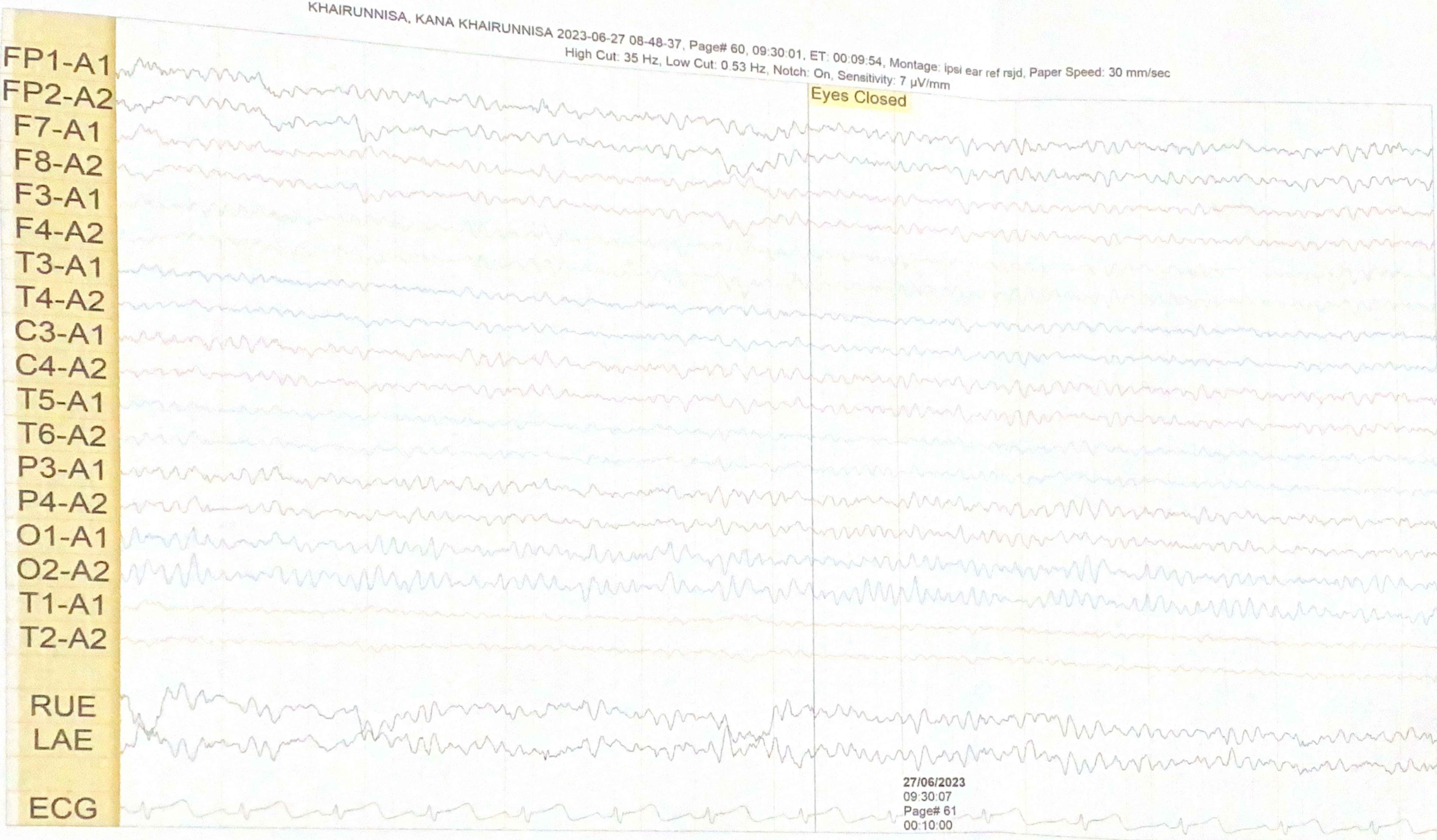

Supplement: Supplementary file 1 [file Data_Sheet_1.pdf]
